# Transcriptomic and metabolomic profiling reveals media- and host-dependent responses to *Staphylococcus hominis* in cell models

**DOI:** 10.7717/peerj.20899

**Published:** 2026-03-12

**Authors:** Wenxiu Liu, Tiansheng Zhang, Qian Wang, Zhunduo Li, Yanrui Bai, Han Xiao, Yan Wang, Ruihong Xiao, Liyu Tong, Yana Li, Xueli Qu, Xu Zhao, Zhengchao Zhang, Hui Sun

**Affiliations:** 1 The Second Hospital & Clinical Medical School, Lanzhou University, Lanzhou, China; 2 Cuiying Biomedical Research Center, The Second Hospital & Clinical Medical School, Lanzhou University, Lanzhou, China; 3 Lanzhou University, Lanzhou, China; 4 Research and Translational Center for Immunological Disorders, Yantai Affiliated Hospital of Binzhou Medical University, Yantai, China; 5 Department of Pathophysiology, School of Basic Medicine, Binzhou Medical University, Yantai, China; 6 Institute of Urban Agriculture, Chinese Academy of Agricultural Sciences, Chengdu, China; 7 Department of Urology, Yantai Affiliated Hospital of Binzhou Medical University, The Second Clinical Medical College of Binzhou Medical University, Yantai, China

**Keywords:** Gut-derived *S. hominis*, BHI and GAM media, Cell line models, Metabolome, RNA sequencing

## Abstract

**Background/aims:**

Host-microbiota co-evolution maintains homeostasis *via* metabolic, immune, and neuroendocrine pathways. Diverse culture media and host cell models are widely used in microbiota research, but how these variables shape host transcriptional responses remains unclear. This study combined metabolomic, transcriptomic, and functional analyses to investigate how microbial culture medium and host cell type influence responses to a gut-derived *Staphylococcus hominis* isolate.

**Methods:**

*S. hominis* was cultured for 96 h in Brain Heart Infusion (BHI) or Gifu Anaerobic Medium (GAM). Culture supernatants were collected for untargeted metabolomics and epithelial cell stimulation. Metabolomic profiling identified differentially expressed metabolites (—log_2_FC— >0, *p* < 0.05, variable importance in projection, VIP >1). RNA sequencing assessed transcriptional responses in four cell lines (MODE-K, NCM460, Henle-407, HEK-293T) treated with BHI- or GAM-derived supernatants. Differentially expressed genes (DEGs; —log_2_FC— >1, adjusted *p* < 0.05) were subjected to Kyoto Encyclopedia of Genes and Genomes (KEGG) pathway enrichment, and principal component and correlation analyses were used to characterize transcriptional changes under different treatments. Functional assays quantified interleukin-6 (IL-6) and interleukin-8 (IL-8) secretion, intracellular triglyceride levels, and the lactate/pyruvate ratio.

**Results:**

Metabolomics revealed medium-dependent remodeling of the exometabolome: BHI-derived supernatants were enriched in fatty acyls, glycerophospholipids, and pathways related to carbohydrate, energy, and lipid metabolism, whereas GAM-derived supernatants contained higher levels of sphingolipids and organic carbonic acids linked to purine and polyunsaturated fatty acid metabolism. Across four cell lines, DEGs induced by BHI-derived supernatants were mainly enriched in metabolic pathways, while GAM-derived supernatants more often engaged immune- and inflammation-related pathways. DEG overlap between cell types was limited, and KEGG enrichment and multivariate analyses supported cell type-specific transcriptional patterns. Functionally, GAM-derived supernatants significantly increased IL-6 and IL-8 secretion, whereas BHI-derived supernatants were more closely associated with changes in intracellular triglycerides and the lactate/pyruvate ratio in a cell-dependent manner (*p* < 0.05).

**Conclusion:**

Metabolomic, transcriptomic, and functional data demonstrate that microbial culture conditions and host cell identity critically shape *in vitro* readouts of host–microbe interactions and should therefore be carefully considered when designing and interpreting microbiota-host interaction studies.

## Introduction

The gut symbiotic microbiota has co-evolved with the host, forming a reciprocal relationship that critically regulates metabolism, immunity, and neurophysiology to maintain host homeostasis (Kim et al., 2023; Sommer & Bäckhed, 2013). When dysbiosis occurs in the gut microbiota, it significantly disrupts metabolic and immune processes, potentially triggering various diseases (Fan & Pedersen, 2021; Levy et al., 2017). To advance the understanding of how intestinal bacteria influence host physiology, researchers have developed a range of culture media informed by culturomics, along with advanced cultivation techniques such as anaerobic intestinal microarrays. In parallel, diverse host models-including immortalized cell lines, intestinal organoids, and germ-free mouse models-have been established to facilitate mechanistic studies (Hirayama, 1999; Jalili-Firoozinezhad et al., 2019; Lagier et al., 2018; Puschhof, Pleguezuelos-Manzano & Clevers, 2021). Despite advances in gut microbiota research, the potential impact of different culture media and host cell models on microbial functional characterization remains insufficiently explored.

Host-microbiota interactions are primarily mediated by microbial metabolites, including vitamins, amino acids, and short-chain fatty acids (SCFAs) (Rooks & Garrett, 2016; Spivak, Fluhr & Elinav, 2022). The composition of culture media significantly influences microbial metabolism by altering bacterial growth dynamics and metabolite production, thereby affecting host responses (Yousi et al., 2019). However, *in vitro* cultured gut microbes often differ substantially from their *in vivo* counterparts, with the choice of culture medium identified as a major determinant of these differences (Tao et al., 2023). Among the commonly used media, BHI and GAM are designed to mimic the intestinal environment and support the growth of anaerobic gut bacteria (Tidjani Alou et al., 2020). Each medium possesses distinct nutritional and physicochemical properties that shape both microbial composition and metabolic output profiles (Giri et al., 2022; Gotoh et al., 2017; Li et al., 2022).

Previous studies have shown that *Clostridium difficile* enhances vitamin and purine synthesis in BHI, while expression of butyrate-associated proteins is reduced in basic media (Otto et al., 2016). In GAM, *E. faecalis* produces high levels of lactic acid, whereas *Bacteroides* and *Parabacteroides* predominantly generate acetate and propionate (Gotoh et al., 2017). Nevertheless, systematic comparisons of how different culture media shape microbial metabolic profiles and their downstream effects on host cells remain limited.

Current cell models for studying gut microbiota functions include immune cells, epithelial cell lines, *etc*, each providing valuable *in vitro* platforms. Immune cells such as macrophages, dendritic cells, and T cells are essential for evaluating microbiota-driven immunoregulation. For example, SCFAs like n-butyrate modulate gut macrophage function and support immune tolerance, as shown using bone marrow-derived macrophages (BMDMs) (Chang et al., 2014). Epithelial cell lines, including NCM460, MODE-K, and Henle-407, are widely used to model microbiota-barrier interactions. The NCM460 line, a non-transformed human colonic epithelial cell type, closely mimics the physiological environment of the colon where gut microbiota predominantly reside (Moyer et al., 1996); The murine duodenal MODE-K, enabling cross-species and regional comparison (Vidal et al., 1993); and Henle-407, a well-established model for bacterial adhesion and invasion (Paton et al., 1997; Zierler & Galán, 1995). Studies have shown that *Faecalibacterium prausnitzii*-derived microbial anti-inflammatory molecules (MAM) restore barrier integrity *via* ZO-1 expression in NCM460 cells (Xu et al., 2020). while *Lactobacillus reuteri* transiently activates MODE-K cells to maintain mucosal homeostasis (Hoffmann et al., 2008). HEK-293T cells are a well-characterized human epithelial-like tool cell line with a defined genetic background, high transfectability, low basal activation, relatively stable signaling, and excellent suitability for the construction of pathway reporter systems, making them a tractable model for dissecting host-microbe interaction pathways (Cohen et al., 2015). It is widely used as a sentinel cell in microbial and immunological studies, particularly for probing NF-κB and NOD-like receptor signaling (Duncan et al., 2017; Inohara et al., 2001), supporting its utility as a validated platform for probing conserved host-pathogen signaling mechanisms. These models differ in function and application, yet systematic comparisons to guide optimal model selection within the same type of cell remain limited.

*S. hominis* is a common member of the human skin microbiota but also an opportunistic pathogen with notable antibiotic resistance, capable of forming persistent biofilms on implanted devices and causing infections such as cellulitis, bacteremia, and infective endocarditis (Uddin et al., 2022; Villarreal-Salazar et al., 2022). In this study, we investigated a strain of *S. hominis* isolated from the human intestinal tract, representing an uncommon ecological niche for this species. Although traditionally considered a skin commensal, emerging evidence suggests its presence in the gut (Leite et al., 2024). This atypical intestinal origin suggests potential gut adaptation, yet the pathogenic and host-interactive properties of gut-derived *S. hominis* remain largely unknown. To begin addressing this gap, we used a gut-derived *S. hominis* strain to explore how bacterial culture conditions and host cell type are associated with epithelial responses, using bacterial culture supernatant metabolomics, multi-cell line transcriptomics, and targeted functional assays. By culturing *S. hominis* in BHI or GAM and exposing four epithelial cell lines (MODE-K, NCM460, Henle-407, HEK-293T) to the resulting supernatants, we observed that both the growth medium and host cell identity were linked to the overall pattern of transcriptional and functional readouts, with culture conditions tending to bias responses toward metabolic *versus* immune/inflammatory programs and each cell line exhibiting a distinct response profile. These observations may help inform the theoretical framework for interpreting microbial functional outputs *in vitro* and underscore the importance of culture environment and host model selection in microbiome research.

## Materials and Methods

### Reagents

BHI medium (Catalog No. CM1135B) was purchased from OXOID (Hampshire, England); GAM medium (Catalog No. 05422) was purchased from Nissui (Tokyo, Japan); Dulbecco’s Eagle Medium, High Glucose (Catalog No. C11995500BT), Fetal Bovine Serum (Catalog No. 16000-044), 0.05% Trypsin-EDTA (1 ×) (Catalog No. 25300-054), Anti-Anti antibiotic solution (100 ×) (Catalog No. 15240-062) were purchased from Gibco (Waltham, MA, USA) Trizol (Catalog No. AG21101) was purchased from Aikore Biological.

### Growth conditions of bacterial strains and genomic sequencing

A *Staphylococcus* strain (named *S. hominis* 327#) is derived from the healthy human gut microbiota repository established in our laboratory. High-pressure sterilized BHI medium was prepared with the following components: brain infusion solids 12.5 g/L, beef heart infusion solids 5.0 g/L, protease peptone 10 g/L, glucose 5 g/L, sodium chloride 5 g/L, disodium phosphate 2.5 g/L, resazurin 1 mg/L, DL-cysteine hydrochloride 1 g/L. Similarly, GAM medium was prepared with peptone 10 g/L, soy peptone 3 g/L, proteopeptide peptone 10 g/L, digestive serum 13.5 g/L, yeast extract 5 g/L, meat extract 2.2 g/L, liver extract 1.2 g/L, glucose 3.0 g/L, potassium dihydrogen phosphate 2.5 g/L, sodium chloride 3.0 g/L, soluble starch 5.0 g/L, L-cysteine hydrochloride 0.3 g/L, thioglycolic acid sodium salt 0.3 g/L, resazurin 1 mg/L, and DL-cysteine hydrochloride 1 g/L. Both media were pre-reduced for at least 48 h in an anaerobic chamber (90% N2: 5% CO2: 5% H2). Inoculate into the culture medium at a ratio of 1:10. Collect the bacteria after growing for two days. The genomic DNA of *S. hominis* was extracted using the E.Z.N.A.^®^ Bacterial DNA Kit (Omega). The genome was then sequenced using a second-generation sequencing platform. Raw reads were quality-corrected and assembled with SPAdes using multiple K-mer sizes to obtain the optimal assembly. Gaps were closed with GapFiller, and fine-scale sequence corrections were performed with PrInSeS-G, resulting in a high-quality draft genome assembly.

### Metabolome sequencing and analysis

For class II liquid samples, aliquots that had been stored at −80 °C were processed as previously described (Li et al., 2024). Briefly, aliquots were thawed on ice and briefly vortexed. Subsequently, 150 µL of extraction solvent (acetonitrile:methanol, 1:4, v/v) containing the internal standard was added to 50 µL of sample. The mixtures were vortexed for 3 min and then centrifuged at 12,000 rpm for 10 min at 4 °C. A 150 µL portion of the resulting supernatant was transferred to a fresh tube and kept at −20 °C for 30 min to further precipitate residual proteins. The samples were centrifuged again at 12,000 rpm for 3 min at 4 °C, and 120 µL of the clarified supernatant was finally transferred into LC-MS vials for analysis.

Quality control (QC) samples were prepared by pooling equal aliquots from all study samples. QC samples were processed using the same extraction protocol as the biological samples and were injected at regular intervals throughout the analytical sequence to monitor instrument stability and analytical reproducibility. Metabolomic analysis was performed by Shanghai Sangon Biotech Co., Ltd. (Shanghai, China).

HPLC and MS settings were adapted from previously described methods (Zhang & Peng, 2025), with minor modifications as follows: For LC-MS analysis, each sample was divided into two aliquots to be analyzed under positive and negative ionization modes. For the positive ion method, chromatographic separation was performed on a Waters ACQUITY Premier HSS T3 column (1.8 µm, 2.1 × 100 mm) at 40 °C. The mobile phases consisted of solvent A (0.1% formic acid in water) and solvent B (0.1% formic acid in acetonitrile). The gradient program was as follows: 5% B at 0 min, linearly increased to 20% B within 1 min, then ramped to 99% B over the next 2 min and held for 1.5 min. The proportion of B was then returned to 5% within 0.1 min and the column was re-equilibrated for 1.4 min. The flow rate was set to 0.4 mL/min and the injection volume was 3 µL. The negative ion method used the same chromatographic conditions and gradient program as the positive ion mode.

MS Conditions (QE): Mass spectrometric detection was performed on a Q Exactive (QE) instrument equipped with an electrospray ionization (ESI) source operated in both positive and negative ion modes. Data acquisition used a data-dependent workflow alternating between full-scan MS and MS/MS (ddMS^2^) with dynamic exclusion. Full-scan spectra were collected over an m/z range of 70–1,000 at a resolution of 60,000 (at m/z 200). The spray voltage was set to 3.8 kV in positive mode and 3.4 kV in negative mode. The sheath gas and auxiliary gas flow rates were 60 and 20 (arbitrary units), respectively. The ion transfer tube and vaporizer temperatures were 320 °C and 350 °C. Normalized collision energies of 30, 40, and 50 eV were applied, and the Top 10 most intense precursor ions were selected for fragmentation in each cycle.

The raw mass spectrometry data were converted into mzML format using ProteoWizard, followed by peak detection, alignment, and retention time correction using the XCMS workflow. Metabolic features with a missing rate >50% within any group were removed. For missing values, a combined imputation strategy was applied: features with >50% missing values were imputed with one-fifth of the minimum detected value, whereas features with <50% missing values were imputed using the K-nearest neighbor (KNN) method. Signal drift correction was performed using support vector regression (SVR). After correction and filtering, metabolite annotation was conducted by matching features against a proprietary in-house metabolite database, supplemented with public spectral libraries and predictive compound databases. Metabolites with a composite identification score ≥ 0.5 and coefficient of variation (CV) <0.3 in QC samples were retained. Data from positive and negative ion modes were subsequently merged, giving priority to metabolites with higher identification confidence and lower CV. Differential metabolites were identified based on VIP (Variable Importance in Projection) values derived from the OPLS-DA model.

### Cell culture and quality control

All cell lines used in this study were kindly provided by the Jorge E. Galán laboratory (Yale University, New Haven, CT, USA). All cells were cultured in high-glucose Dulbecco’s Modified Eagle Medium (DMEM) supplemented with 10% fetal bovine serum (FBS), 100 U/mL penicillin, and 100 µg/mL streptomycin at 37 °C in a humidified atmosphere containing 5% CO_2_. Upon receipt, cells were expanded and cryopreserved as early-passage frozen stocks, and all experiments were performed within a restricted passage window. For human cell lines (NCM460, Henle-407, and HEK-293T), short tandem repeat (STR) profiling was conducted prior to the experiments by an accredited service provider (Gene Carer Biotech Co., Ltd., Xi’an, China). MODE-K was obtained directly from the same laboratory, and its provenance together with stable growth characteristics was used to support its identity. All cell cultures were tested for mycoplasma contamination before experiments using the MycoBlue Mycoplasma Detector kit (Cat. No. D101-01, Vazyme, Shanghai, China) according to the manufacturer’s instructions, and all tested cultures were confirmed to be mycoplasma-negative.

### Bacterial supernatant collection and cell treatment

After reviving *S. hominis*, the culture was incubated for 24 h and then subcultured at a 1:100 ratio into BHI and GAM media for an additional 4 days. The culture supernatant was collected by centrifuging at 10,000 rpm for 10 min at 4 °C, followed by filtration through a 0.22 µm membrane. The collected supernatant from *S. hominis* cultured in BHI/GAM media was used to incubate Henle-407, NCM460, HEK-293T, and MODE-K cells for 5 h. After incubation, the supernatant was discarded and the cells were collected.

### Transcriptome sequencing and analysis

#### RNA extraction and library preparation

Total RNA was extracted using TRIzol reagent following the manufacturer’s instructions. RNA purity and concentration were assessed using a NanoDrop 2000 spectrophotometer (Thermo Scientific, Waltham, MA, USA), and RNA integrity was evaluated using an Agilent 2100 Bioanalyzer (Agilent Technologies, Santa Clara, CA, USA). The transcriptome library was constructed according to the manufacturer’s protocol using the VAHTS Universal V5 RNA-seq Library Prep Kit. Transcriptome sequencing and analysis were performed by Shanghai OE Biotech Co., Ltd. (Shanghai, China).

#### RNA sequencing and data processing

The libraries were sequenced on an Illumina NovaSeq 6000 platform, producing 150 bp paired-end reads. Raw reads in FASTQ format were processed using the fastp (Chen et al., 2018) to filter out low-quality reads. The HISAT2 software (Kim, Langmead & Salzberg, 2015) was used for reference genome alignment, and gene expression levels (FPKM) (Roberts et al., 2011) were calculated. Gene-leve read counts were obtained using HTSeq-count (Anders, Pyl & Huber, 2015). Differential expression analysis was performed using DESeq2 (Love, Huber & Anders, 2014).

#### Power analysis calculation

The statistical power of this RNA-Seq experimental design was calculated using the RNASeqPower tool (https://rodrigo-arcoverde.shinyapps.io/rnaseq_power_calc/). Parameters used for the analysis were: sequencing depth of 50 million reads per sample, coefficient of variation (CV) = 0.2, expected log2 fold change = 2, alpha = 0.05, and a target power of 0.84. The result showed that at least 2.18 biological replicates per group are required to achieve this statistical power.

#### Biological and technical replicates

In this study, three biological replicates were included per group, each representing an independent biological sample. No technical replicates were used.

### Enzyme-linked immunosorbent assay

To assess cytokine production, cells were stimulated with bacterial culture supernatants. Specifically, *S. hominis* was grown in either BHI or GAM medium, after which the culture supernatants were collected, sterilized by filtration (0.22 µm), and applied to NCM460, MODE-K, Henle-407, and HEK-293T cells at a final concentration of 20% (v/v). Following 24 h of stimulation, the cell culture supernatants were harvested and centrifuged at 1,500 × g for 10 min to remove residual particulates. Cytokine concentrations in cell supernatants were measured by enzyme-linked immunosorbent assay (ELISA) according to the manufacturer’s instructions; mouse IL-6 (Cat No: 431307, BioLegend), human IL-8 (Cat#SEKH-0016, Solarbio, China).

### Intracellular triglyceride quantification

Triglyceride levels were measured using a commercial Triglyceride Assay Kit (Beyotime, Shanghai, China) according to the manufacturer’s instructions. All procedures were performed strictly following the kit protocol, and absorbance was recorded using a multifunctional microplate spectrophotometer at the designated wavelength. Triglyceride concentrations were calculated based on the standard curve and compared among samples.

### Lactate/pyruvate ratio measurement

Lactate and pyruvate levels were measured using commercial assay kits (Lactate Assay Kit and Pyruvate Assay Kit, Beyotime, China) in accordance with the manufacturer’s instructions. Lactate concentrations were determined using a microplate reader, while pyruvate concentrations were measured using a multifunctional microplate spectrophotometer. The lactate/pyruvate ratio was calculated by dividing the lactate concentration by the pyruvate concentration.

### Statistical analysis

Bioinformatic analyses were performed using the OECloud platform (https://cloud.oebiotech.com/task/) in combination with R, largely following previously published protocols (Bai et al., 2026). For RNA-seq data, differential expression was estimated with the DESeq2 package, which models read counts using a negative binomial distribution. Wald tests were applied to evaluate pairwise contrasts, and the resulting *p* values were adjusted for multiple testing by the Benjamini–Hochberg procedure to control the false discovery rate (FDR). Genes with an adjusted *q* value < 0.05 and a fold change > 2 or < 0.5 were considered differentially expressed. Functional interpretation of DEGs was performed by KEGG pathway enrichment analysis using the clusterProfiler package. Over-representation of DEGs in each pathway was assessed with hypergeometric tests, and enrichment was summarized as the ratio between the observed and expected numbers of DEGs in a given pathway. Plots, including dot plots, were generated in R (https://www.r-project.org/) with ggplot2 and exported either locally or *via* the OECloud web interface. Multi-cluster fold-change profiles were visualized using the SRplot online platform for data visualization (Tang et al., 2023). For untargeted metabolomics, pairwise comparisons of metabolite abundances between groups were performed using Student’s t tests, and multivariate importance was evaluated with an OPLS-DA model. Metabolites satisfying —log_2_(fold change)—>0, a VIP score >1.0, and *p* < 0.05 were defined as differentially expressed metabolites (DEMs) contributing to group separation. Identified metabolites were first annotated against the KEGG Compound database (http://www.kegg.jp/kegg/compound/), and the annotated compounds were subsequently mapped to pathways in the KEGG Pathway database (http://www.kegg.jp/kegg/pathway.html) for enrichment analysis.

For all functional assays, including ELISA-based cytokine measurements, intracellular triglyceride quantification, and lactate/pyruvate ratio analyses, each experimental condition included at least two biologically independent replicates (independent cell cultures treated in parallel) and two technical replicates (duplicate wells per condition within each experiment). Data are presented as mean ± SEM. Statistical analyses were performed using one-way ANOVA followed by Tukey’s *post-hoc* test (GraphPad Prism v9). A *p*-value <0.05 was considered statistically significant. Exact *p* values are reported in the figure legends.

## Results

### Genome sequencing, metabolomic and transcriptome quality control analysis

To clarify whether the supernatants of *S. hominis* cultured in different media have differential effects on different cell lines, we first conducted whole-genome sequencing of the opportunistic pathogen *S. hominis* (Fig. S1A). Then, we collected the supernatants of *S. hominis* cultured in BHI and GAM for 96 h, using the corresponding media as controls, and first subjected these supernatants to metabolomic analysis. Subsequently, the supernatants were used to treat MODE-K, NCM460, Henle-407, and HEK-293T cells, after which transcriptome sequencing was performed on the treated cells (Fig. S1B).

To assess the quality of the metabolomic data, Pearson correlation analysis was performed among QC samples in both negative and positive ion modes. In the negative ion mode, all pairwise correlations between QC injections were close to 1 (—r— ≥ 0.999) (Fig. S1C), and similarly high correlations were observed in the positive ion mode (Fig. S1D). These results indicate excellent consistency among QC runs and demonstrate that the analytical system exhibited high reproducibility and stability, ensuring that the metabolomic data are reliable for subsequent differential metabolite analysis.

A total of 24 samples were subjected to reference-based transcriptome sequencing. Clean data output ranged from 6.87 to 7.06 GB per sample, with Q30 scores between 94.32% and 97.48% and an average GC content of 49.97%. Reads were aligned to the corresponding reference genomes using HISAT2 (Kim, Langmead & Salzberg, 2015), with alignment rates between 95.55% and 97.98% (Tables S1–S2). Protein-coding gene expression was quantified using HTSeq-count (Anders, Pyl & Huber, 2015) and genes with zero expression across all samples were excluded from downstream analyses. The number of genes detected in each sample is shown in (Figs. S1E, S1F).

### Comparative metabolomic profiling of BHI- and GAM-derived *S. hominis* supernatants

**Figure 1:**
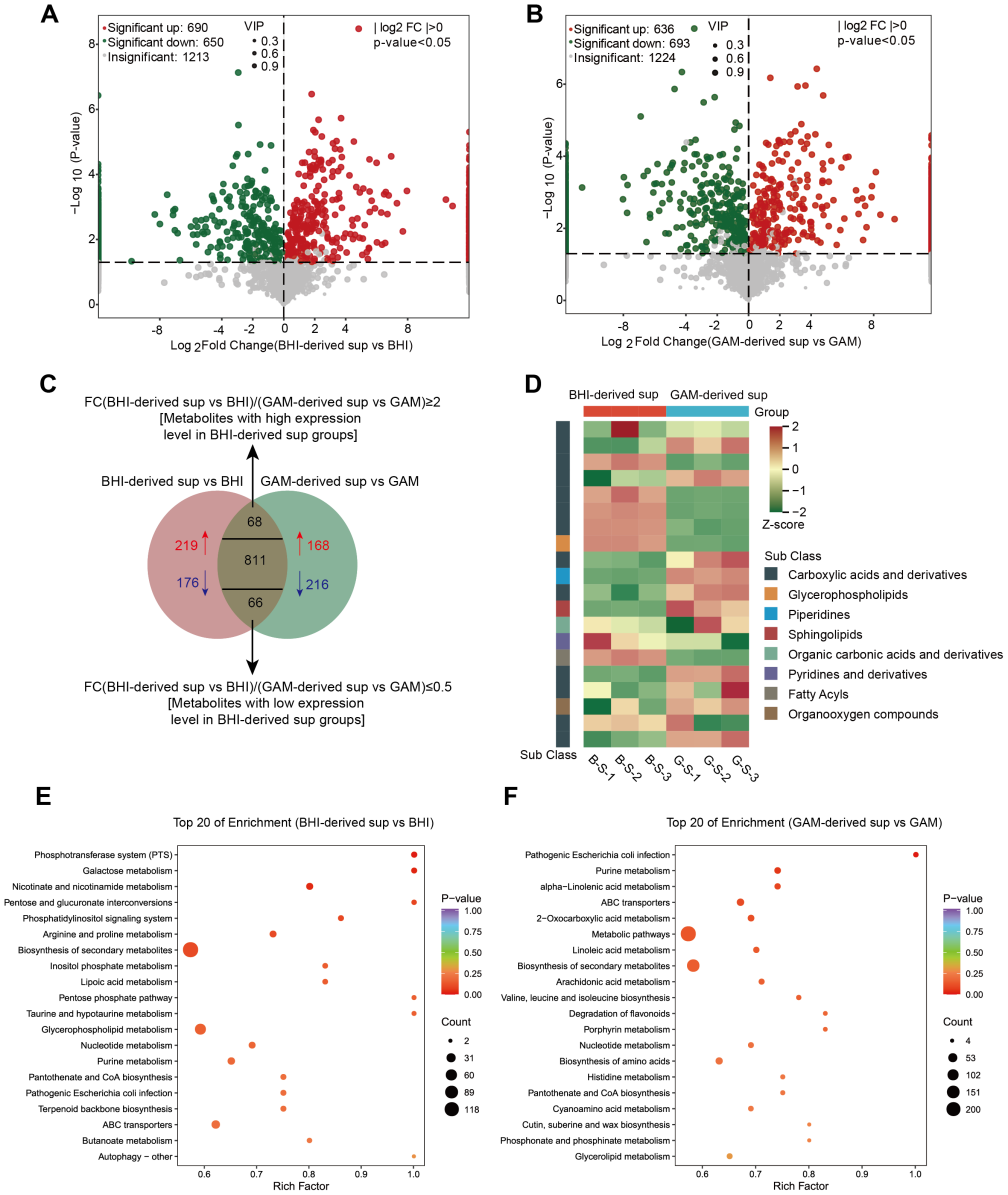
Metabolomic profiling of *S. hominis* supernatants under different culture conditions. (A) Volcano plot of differentially expressed metabolites (DEMs) in the BHI-derived *S. hominis* supernatant *versus* BHI control, *n* = 3 per group. Red dots indicate significantly upregulated metabolites, green dots indicate significantly downregulated metabolites, and gray dots represent metabolites with no significant changes (|log_2_FC| > 0, *p* < 0.05, VIP > 1). Dot size corresponds to the VIP value. (B) Volcano plot of DEMs in the GAM-derived *S. hominis* supernatant *versus* GAM control. Color coding and VIP value representation are the same as in panel (A). (C) Venn diagram illustrating overlapping and unique DEMs between the BHI-derived *S. hominis* supernatant and GAM-derived *S. hominis* supernatant groups relative to the medium control group. (D) Heatmaps display the top 20 significantly altered metabolites between the two conditions. Colors indicate normalized metabolite abundance after unit variance (UV) scaling: red corresponds to higher levels and green to lower levels. Metabolite classification was annotated according to HMDB subclass (Class), with color codes representing different chemical categories. (E–F) KEGG pathway enrichment analysis of the top 20 enriched pathways in the BHI-derived *S. hominis* supernatant group (E) and the GAM-derived *S. hominis* supernatant group (F), each compared with the corresponding medium control. The Rich Factor represents the ratio of the number of differential metabolites to the total number of metabolites annotated to a given pathway, with higher values indicating a greater degree of enrichment. The *P*-value was calculated using a hypergeometric test, and the top 20 pathways were selected and ranked in ascending order of *P*-value for visualization.

To compare the metabolic profiles of *S. hominis* cultured under different nutritional conditions, we performed untargeted metabolomic analysis on supernatants collected from BHI- and GAM-grown bacteria. Volcano plot analysis showed numerous differential metabolites in both conditions (Figs. 1A–1B). Relative to the BHI medium control, the BHI-derived *S. hominis* supernatant contained 690 upregulated and 650 downregulated metabolites (—log_2_FC— > 0, *p* < 0.05, VIP > 1) (Fig. 1A). Similarly, the GAM-derived *S. hominis* supernatant contained 636 upregulated and 693 downregulated metabolites relative to the GAM control (Fig. 1B). Venn analysis identified 811 differential metabolites shared between both conditions (Fig. 1C). In addition, the BHI-derived *S. hominis* supernatant included 219 uniquely upregulated and 176 uniquely downregulated metabolites, while the GAM-derived *S. hominis* supernatant included 168 uniquely upregulated and 216 uniquely downregulated metabolites, indicating differences in metabolite composition between the two culture conditions.

As shown in the heatmap, visualization of the top 20 significantly altered metabolites revealed distinct subclass patterns between the two culture conditions. Fatty acyls and glycerophospholipids were mainly associated with the BHI-derived *S. hominis* supernatant, whereas sphingolipids and organic carbonic acids and derivatives were more enriched under the GAM condition. These patterns are consistent with the overall metabolic distinctions identified between the two culture environments (Fig. 1D). KEGG pathway enrichment analysis revealed condition-specific metabolic patterns in the *S. hominis* supernatants (Figs. 1E–1F). In the BHI-derived *S. hominis* supernatant, differential metabolites were mainly associated with carbohydrate and energy metabolism pathways, including the phosphotransferase system (PTS), galactose metabolism, and nicotinate and nicotinamide metabolism, as well as several amino acid- and lipid-related pathways such as arginine and proline metabolism and glycerophospholipid metabolism. The GAM-derived *S. hominis* supernatant was characterized by enrichment in nucleotide, fatty acid, and amino acid metabolism, including purine metabolism, α-linolenic acid metabolism, arachidonic acid metabolism, and valine, leucine, and isoleucine biosynthesis. Overall, these analyses suggest that the extracellular metabolite profiles of *S. hominis* differ depending on the culture medium.

### Comparative transcriptomic profiles of MODE-K cell treated with distinct *S. hominis* culture supernatants

**Figure 2:**
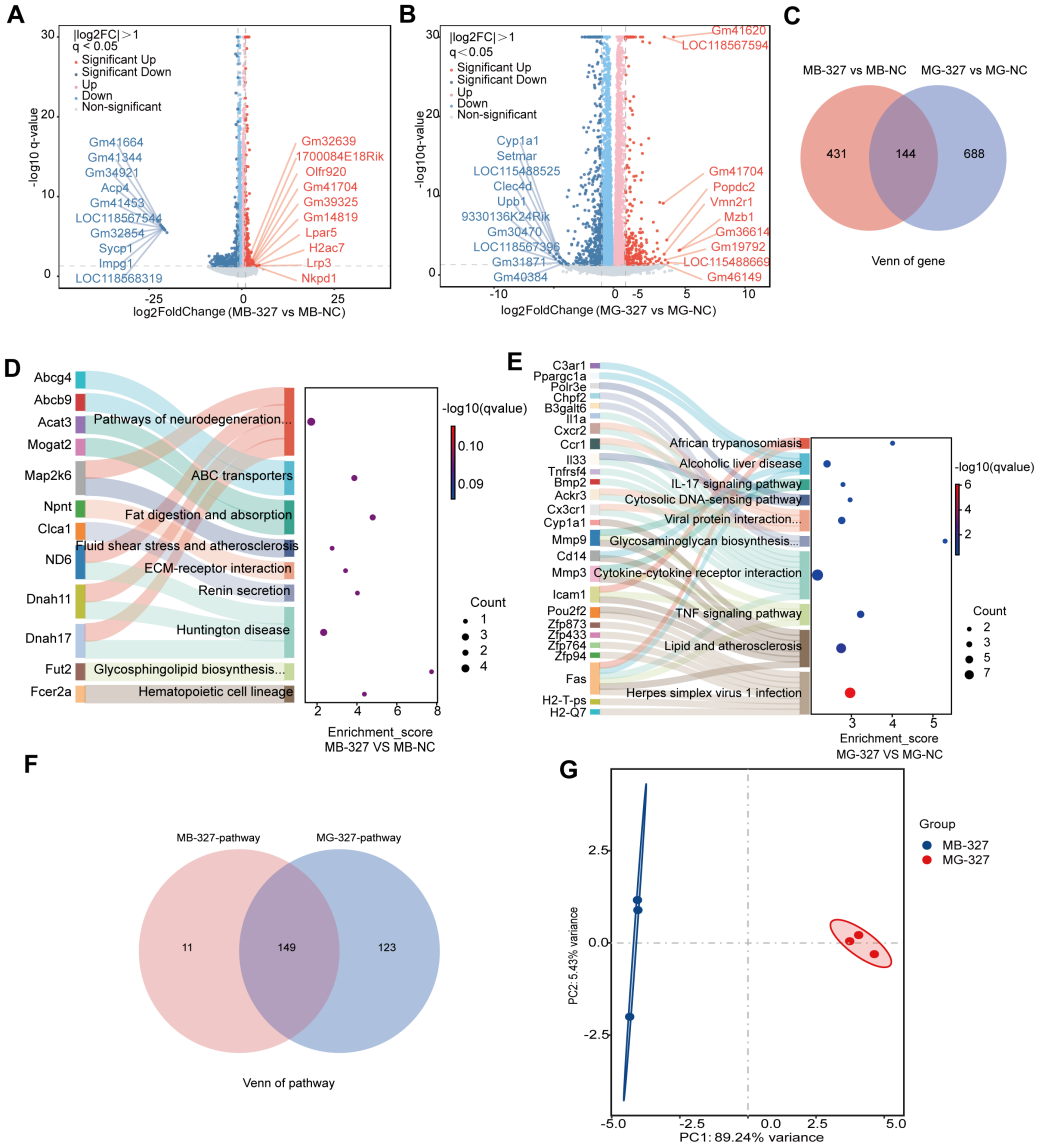
Transcriptomic changes in MODE-K cell line. (A) Volcano plots of differentially expressed genes (DEGs) in the MODE-K cell line of the supernatant of *S. hominis* cultured by BHI (MB-327) compared to the control group (MB-NC), *n* = 3 in per group. Red dots indicate significantly upregulated genes, blue dots indicate significantly downregulated genes, and gray dots represent genes with no significant changes. Key DEGs are annotated. (B) Volcano plot of DEGs in the MODE-K cell line of the supernatant of *S. hominis* cultured by GAM (MG-327) compared to the MG-NC. Color coding is the same as in panel (A), with annotated genes indicating key DEGs. (C) Venn diagram showing overlapping and unique DEGs between the MB-327 and MG-327 groups compared to the NC group. (D–E) Sankey bubble plot of top 10 pathways enriched in the transcriptome of the MB-327 group (D) and in MG-327 group (E). The left panel lists enriched pathways, the right panel shows the associated genes. (F) Venn diagram showing overlapping and unique KEGG-enriched pathways between the MB-327 and MG-327 groups compared to the NC group. (G) Principal component analysis (PCA) of transcriptomic datasets, illustrating group separation. Differentially expressed genes were identified using DESeq2 with |log_2_FC| > 1 and FDR < 0.05 as the significance threshold.

Volcano plot analysis, executed through DESeq2 (—log2FC— > 1, adjusted *p* < 0.05), elucidated differential gene expression profiles between the NC group and the *S. hominis* supernatant treatment groups (Figs. 2A and 2B). In the MB-327 group, 575 DEGs were identified, whereas the MG-327 group demonstrated 832 DEGs compared to the NC. Comparative analysis showed that there were 431 and 688 unique genes in MB-327 group and MG-327 group respectively, and there was only a 144-gene overlap between the two groups (Fig. 2C). These results indicate that the transcriptional programs associated with the MB-327 and MG-327 group are largely different, reflecting specific biological responses.

To compare the functional impact of BHI- *versus* GAM-derived *S. hominis* supernatants on mouse MODE-K cells, KEGG pathway analysis was performed for MB-327 and MG-327 groups based on significantly regulated DEGs (Figs. 2D–2E), focusing on the top 10 enriched pathways. In the MB-327 group, DEGs were significantly enriched in pathways associated with neurodegeneration (*e.g.*, Huntington disease), fat digestion and absorption, fluid shear stress and atherosclerosis, ECM-receptor interaction, and vascular remodeling. These findings suggest that BHI-cultured *S. hominis* supernatant may influence consistent with prominent remodeling of lipid handling, extracellular matrix signaling. In contrast, MG-327 DEGs were predominantly enriched in immune-related pathways, such as cytokine–cytokine receptor interaction, TNF signaling, IL-17 signaling, and herpesvirus infection. This indicates that GAM-cultured supernatant is associated with a stronger innate immune and inflammatory response. Together, these findings suggest that culture conditions significantly influence the functional impact of *S. hominis* supernatants.

To further compare the functional impacts of BHI- and GAM-derived *S. hominis* supernatants, we analyzed KEGG pathway overlaps using a Venn diagram (Fig. 2F). While 149 pathways were shared, MB-327 and MG-327 groups exhibited 11 and 123 unique pathways, respectively. This indicates that GAM-cultured supernatant is associated with a broader and more diverse transcriptional response. To further dissect the biological differences and similarities between MB-327 and MG-327 groups, KEGG pathway enrichment analysis was categorized into group-specific and shared pathways (Fig. S2). Shared enriched pathways between the two groups were mainly involved in immune system and infectious disease processes, suggesting a common immune activation background with divergent pathway profiles. These findings indicate that MB-327 group is primarily linked to metabolic and vascular responses, while MG-327 group shows a dominant immune and inflammatory signature, with both groups sharing core immune-related processes.

Principal component analysis (PCA) was conducted to assess the global transcriptional profiles of MB-327 group and MG-327 group. The two groups were distinctly separated along the first principal component (PC1), which accounted for 89.24% of the total variance, while PC2 explained 5.43%. The clear separation along PC1 indicates substantial differences in gene expression patterns between MB-327 group and MG-327 group. Furthermore, tight clustering within each group suggests high reproducibility and strong intra-group consistency (Fig. 2G). These findings suggest that different bacterial culture conditions (BHI *vs.* GAM) may alter the functional properties of *S. hominis*, leading to distinct transcriptional responses in host cells. This highlights the importance of microbial environmental context in shaping host-microbe interactions.

### Comparative transcriptomic profiles of NCM460 cell treated with distinct *S. hominis* culture supernatants

**Figure 3:**
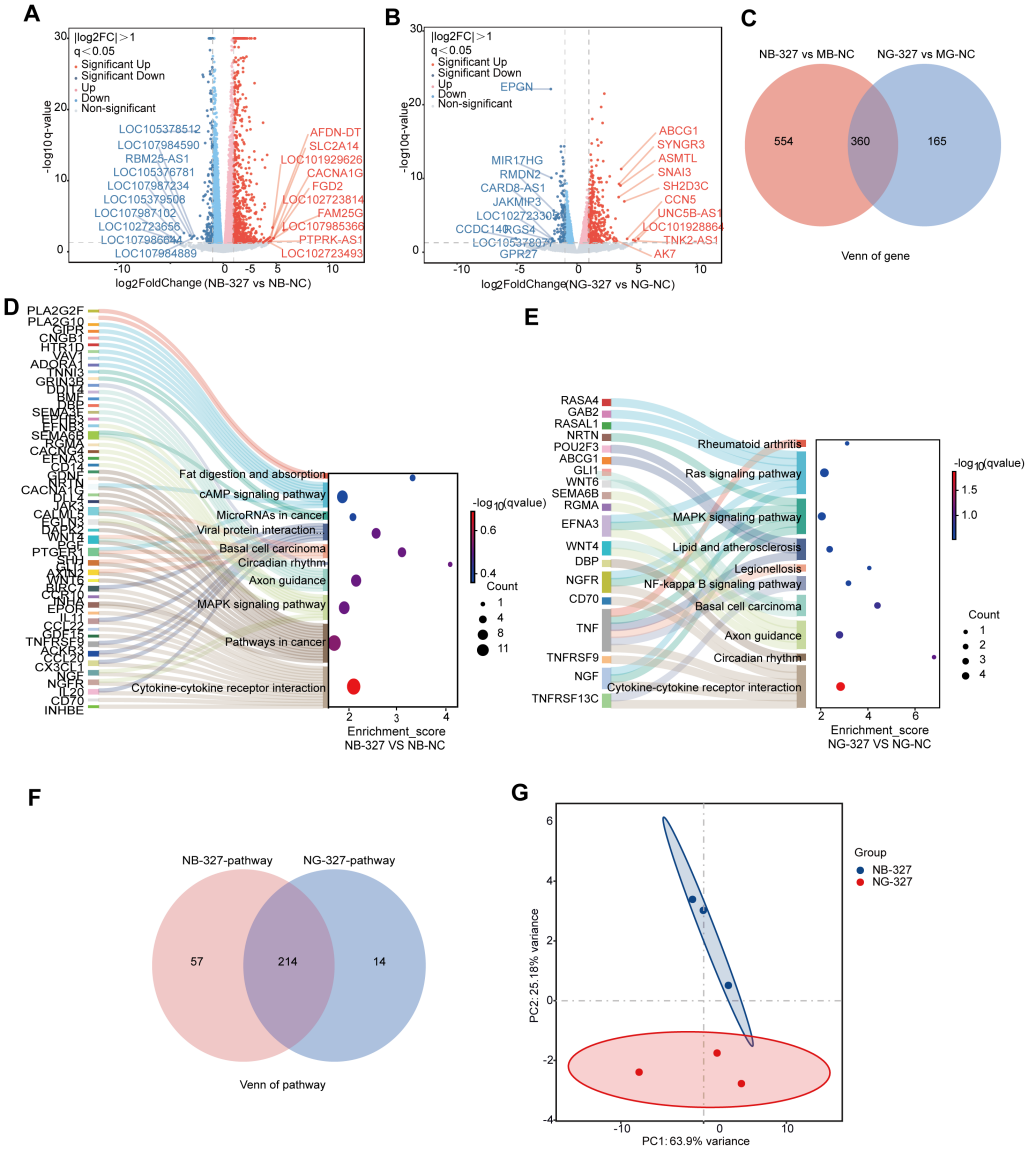
Transcriptomic changes in NCM460 cell line. (A) Volcano plots of DEGs in the NCM460 cell line of the supernatant of *S. hominis* cultured by BHI (NB-327) compared to the control group (NB-NC), *n* = 3 in per group. Red dots indicate significantly upregulated genes, blue dots indicate significantly downregulated genes, and gray dots represent genes with no significant changes. Key DEGs are annotated. (B) Volcano plot of DEGs in the NCM460 cell line of the supernatant of *S. hominis* cultured by GAM (NG-327) compared to the NG-NC. Color coding is the same as in panel (A), with annotated genes indicating key DEGs. (C) Venn diagram showing overlapping and unique DEGs between the NB-327 and NG-327 groups compared to the NC group. Heatmaps display the expression profiles of significantly altered genes between the NB-327 and NG-327 groups, visually capturing the expression patterns of the transcriptome. (D–E) Sankey bubble plot of top 10 pathways enriched in the transcriptome of the NB-327 group (D) and in NG-327 group (E). The left panel lists enriched pathways, the right panel shows the associated genes. (F) Venn diagram showing overlapping and unique KEGG-enriched pathways between the NB-327 and NG-327 groups compared to the NC group. (G) PCA of transcriptomic datasets, illustrating group separation. Differentially expressed genes were identified using DESeq2 with |log_2_FC| > 1 and FDR < 0.05 as the significance threshold.

To extend and validate our findings, we analyzed the transcriptomic responses of human colonic epithelial NCM460 cells exposed to *S. hominis* supernatants cultured in BHI or GAM media. Volcano plot analysis, executed through DESeq2 (—log2FC— > 1, adjusted *p* < 0.05), elucidated differential gene expression profiles between the NC group and the *S. hominis* supernatant treatment groups (Figs. 3A and 3B). In the NB-327 group, 914 DEGs were identified, whereas the NG-327 group demonstrated 525 DEGs compared to the NC. Venn diagram analysis revealed that NB-327 group and NG-327 group shared 360 DEGs, while 554 were unique to NB-327 group, and 165 were unique to NG-327 group (Fig. 3C). These results suggest that the two treatments elicit distinct transcriptional profiles, with a small core set of overlapping gene responses.

KEGG pathway enrichment analysis revealed distinct functional profiles in NB-327 and NG-327 groups (Figs. 3D and 3E). DEGs were enriched in pathways related to lipid metabolism (*e.g.*, fat digestion and absorption), MAPK signaling, and oncogenic regulation such as microRNAs in cancer, indicating potential metabolic and proliferative changes in NB-327 group. In contrast, NG-327 group showed enrichment in immune and inflammatory pathways, including, Ras and MAPK signaling, NF-κB signaling, cytokine-cytokine receptor interaction. These findings suggest that while both groups engage cellular signaling networks, NB-327 is associated with metabolic and signaling regulation, whereas NG-327 more strongly is associated with inflammatory and host-pathogen interaction pathways. To further compare the functional similarities and differences between NB-327 group and NG-327 group, we examined the overlap in enriched KEGG pathways. Venn diagram analysis of KEGG pathway enrichment revealed that NB-327 and NG-327 groups shared 214 common pathways, indicating a large degree of overlap in their biological responses. However, 57 unique enrichment pathways were exhibited in the NB-327 group, while 14 were shown in the NG-327 group (Fig. 3F). This indicates that the NB-327 group may induce broader or more diverse pathway activations. These results highlight both common and group-specific molecular mechanisms underlying their respective transcriptomic responses. To delineate functional differences between NB-327 and NG-327 group, KEGG pathways were categorized as group-specific or shared (Fig. S3). NB-327-specific pathways were enriched in metabolic and biosynthetic processes, including amino acid metabolism and glycosphingolipid biosynthesis. In contrast, NG-327 showed enrichment in immune-related pathways such as RIG-I-like receptor signaling and graft-versus-host disease. Shared pathways (*e.g.*, NF-kappa B and MAPK signaling) reflected common immune activation. These findings indicate that BHI-cultured *S. hominis* supernatant mainly modulates metabolism, while GAM-cultured supernatant is associated with stronger immune responses.

PCA was conducted to assess the global transcriptional profiles of NB-327 and NG-327 groups (Fig. 3G). In the transcriptomic dataset, PC1 and PC2 accounted for **63.9%** and **25.1%** of the total variance, respectively. The clear separation along PC1 indicates substantial differences in gene expression patterns between NB-327 group and NG-327 group. Furthermore, tight clustering within each group suggests high reproducibility and strong intra-group consistency. The above results indicate that after treating NCM460 cells with the *S. hominis* supernatant cultured in BHI and GAM media, there are also significant differences in the differential gene expression profiles and KEGG enrichment pathways.

### Comparative transcriptomic profiles of Henle-407 cell treated with distinct *S. hominis* culture supernatants

**Figure 4:**
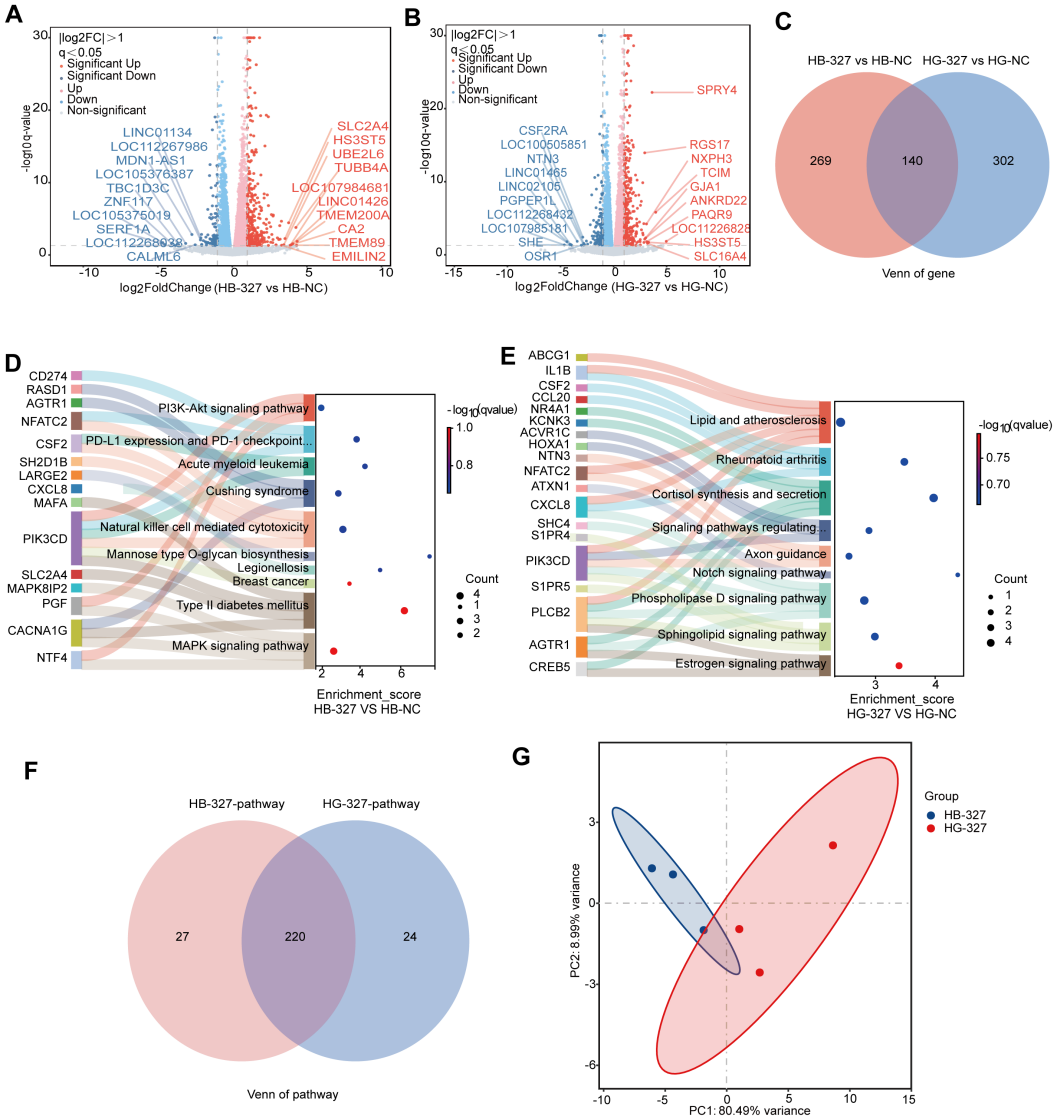
Transcriptomic changes in Henle-407 cell line. (A) Volcano plots of DEGs in the Henle-407 cell line of the supernatant of *S. hominis* cultured by BHI (HB-327) compared to the control group (HB-NC), *n* = 3 in per group. Red dots indicate significantly upregulated genes, blue dots indicate significantly downregulated genes, and gray dots represent genes with no significant changes. Key DEGs are annotated. (B) Volcano plot of DEGs in the Henle-407 cell line of the supernatant of *S. hominis* cultured by GAM (HG-327) compared to the HG-NC. Color coding is the same as in panel (A), with annotated genes indicating key DEGs. (C) Venn diagram showing overlapping and unique DEGs between the HB-327 and HG-327 groups compared to the NC group. Heatmaps display the expression profiles of significantly altered genes between the HB-327 and HG-327 groups, visually capturing the expression patterns of the transcriptome. (D–E) Sankey bubble plot of top 10 pathways enriched in the transcriptome of the HB-327 group (D) and in HG-327 group (E). The left panel lists enriched pathways, the right panel shows the associated genes. (F) Venn diagram showing overlapping and unique KEGG-enriched pathways between the HB-327 and HG-327 groups compared to the NC group. (G) PCA of transcriptomic datasets, illustrating group separation. Differentially expressed genes were identified using DESeq2 with |log_2_FC| > 1 and FDR < 0.05 as the significance threshold.

To investigate whether the supernatant of *S. hominis* cultured in BHI or GAM also had different effects on another type of human intestinal epithelial cell, Henle-407. To identify specific transcriptomic changes, we conducted differential gene expression analysis comparing each treatment group to its corresponding control. Volcano plot analysis, executed through DESeq2 (—log2FC— > 1, adjusted *p* < 0.05), revealed distinct sets of significantly up- and downregulated genes in HB-327 and HG-327 groups compared to their respective controls (Figs. 4A and 4B). In the HB-327 group, 409 DEGs were identified, whereas the HG-327 group demonstrated 442 DEGs compared to the NC. A comparative Venn diagram and heatmap analysis revealed that HB-327 and HG-327 groups shared only 140 differentially expressed genes, while 269 and 302 DEGs were unique to HB-327 and HG-327 groups, respectively (Fig. 4C). This again highlights the distinct transcriptional responses was associated with treatment condition.

To explore functional implications, we performed KEGG pathway enrichment analysis on significantly regulated DEGs. DEGs were significantly enriched in PI3K-Akt signaling, MAPK signaling, natural killer cell-mediated cytotoxicity, and PD-L1 expression and checkpoint pathways in HB-327 group (Fig. 4D), indicating modulation of immune regulation and intracellular signaling. Meanwhile, in HG-327 group, DEGs were enriched in sphingolipid signaling, Notch signaling, and lipid and atherosclerosis pathways (Fig. 4E), suggesting involvement of inflammatory and lipid metabolic processes.

A Venn diagram comparison of KEGG pathways further showed that HB-327 and HG-327 groups shared 220 common pathways, but also retained 27 and 24 unique enriched pathways, respectively (Fig. 4F). These findings suggest the existence of both shared core biological responses and distinct regulatory networks specific to each condition. To further compare HB-327 and HG-327 groups, enriched KEGG pathways were grouped as specific or shared (Fig. S4). HB-327-specific pathways involved amino acid metabolism, steroid biosynthesis, and immune-related processes. HG-327-specific pathways included circadian rhythm, axon guidance, and ferroptosis, indicating neural and stress-related responses. Shared pathways, such as TNF and Notch signaling, reflected common inflammatory and developmental programs.

PCA was first performed to evaluate the overall transcriptional variation across treatment groups. As shown in Fig. 4G, HB-327 and HG-327 samples were clearly separated along PC1, which explained 80.49% of the total variance, with PC2 contributing an additional 8.99%. This distinct segregation indicates that the two treatments was associated with divergent global gene expression profiles, with strong intra-group consistency.

### Comparative transcriptomic profiles of HEK-293T cell treated with distinct *S. hominis* culture supernatants

**Figure 5:**
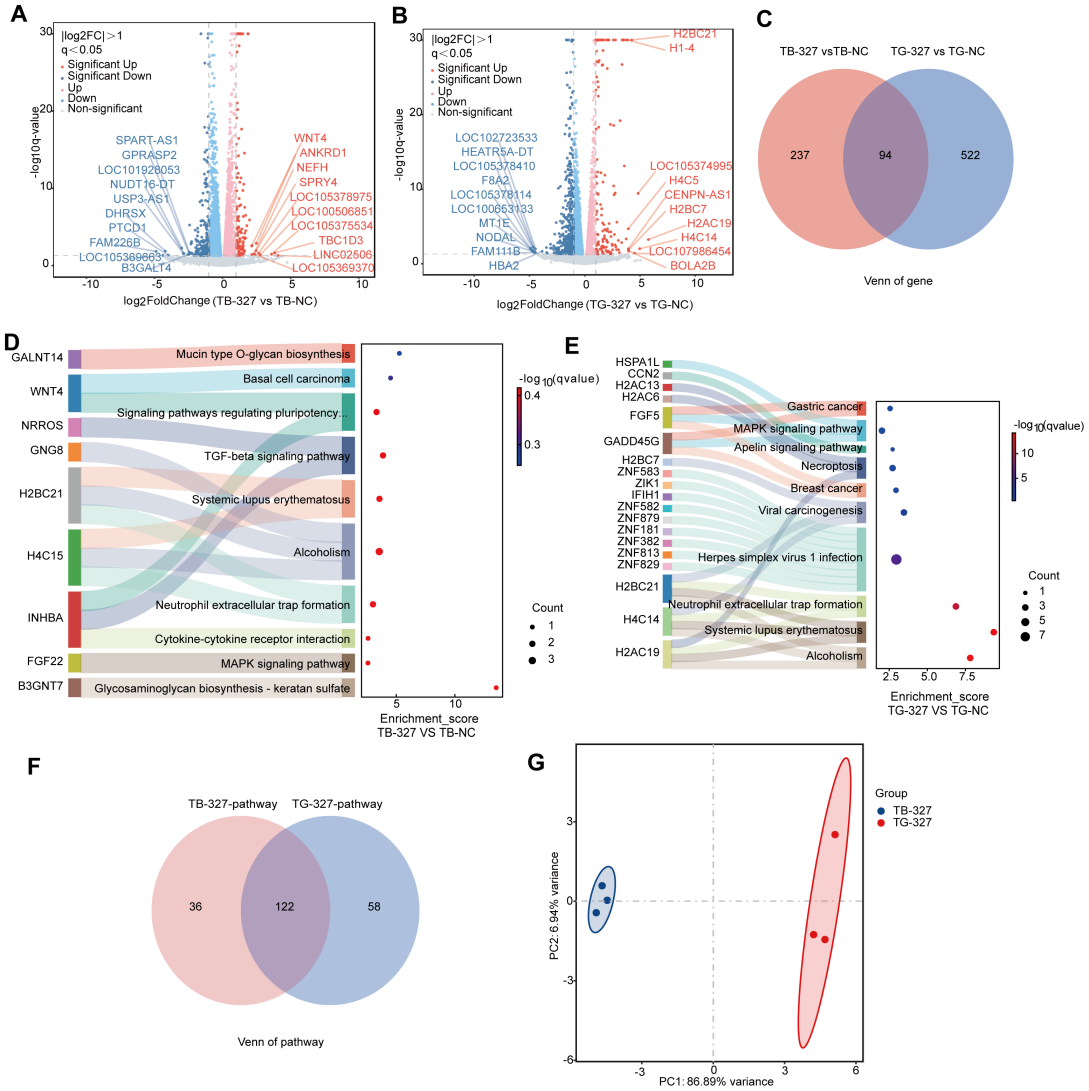
Transcriptomic changes in HEK-293T cell line. (A) Volcano plots of DEGs in the HEK-293T cell line of the supernatant of *S. hominis* cultured by BHI (TB-327) compared to the control group (TB-NC), *n* = 3 in per group. Red dots indicate significantly upregulated genes, blue dots indicate significantly downregulated genes, and gray dots represent genes with no significant changes. Key DEGs are annotated. (B) Volcano plot of DEGs in the HEK-293T cell line of the supernatant of *S. hominis* cultured by GAM (TG-327) compared to the TG-NC. Color coding is the same as in panel (A), with annotated genes indicating key DEGs. (C) Venn diagram showing overlapping and unique DEGs between the TB-327 and TG-327 groups compared to the NC group. Heatmaps display the expression profiles of significantly altered genes between the TB-327 and TG-327 groups, visually capturing the expression patterns of the transcriptome. (D–E) Sankey bubble plot of top 10 pathways enriched in the transcriptome of the TB-327 group (D) and in TG-327 group (E). The left panel lists enriched pathways, the right panel shows the associated genes. (F) Venn diagram showing overlapping and unique KEGG-enriched pathways between the TB-327 and TG-327 groups compared to the NC group. H PCA of transcriptomic datasets, illustrating group separation. Differentially expressed genes were identified using DESeq2 with |log_2_FC| > 1 and FDR < 0.05 as the significance threshold.

Given the distinct transcriptional responses observed in intestinal epithelial cells, we further examined how *S. hominis* supernatants cultured in BHI or GAM affect gene expression in HEK-293T cells-an epithelial line of renal origin-using transcriptome-wide analysis including differential expression, pathway enrichment, and dimensionality reduction. Volcano plot analysis, performed using DESeq2 (—log_2_FC— > 1, adjusted *p* < 0.05), identified statistically significant gene expression changes in each group compared to controls (Figs. 5A and 5B). In TB-327 group, a total of 331 DEGs with were detected. In TG-327 group, 616 DEGs met the same threshold. Venn analysis of DEGs showed only 94 overlapping genes between TB-327 and TG-327 groups, while 237 and 522 genes were unique to each group, respectively (Fig. 5C). The corresponding heatmap illustrated consistent and group-specific expression trends, confirming treatment-specific transcriptional programs.

To investigate the biological processes associated with these DEGs, KEGG pathway enrichment analysis was performed using significantly regulated genes (*p* < 0.05, Benjamini–Hochberg correction). In TB-327 group, enriched pathways were associated with mucin-type O-glycan biosynthesis, glycosaminoglycan biosynthesis (keratan sulfate), MAPK signaling, TGF-β signaling. These patterns suggest prominent remodeling of glycan synthesis and immune-regulatory signaling under BHI-derived supernatant (Fig. 5D). TG-327 group DEGs were enriched in MAPK and Apelin signaling pathways, neutrophil extracellular trap formation, indicating that GAM-derived supernatants was associated with cell-death-, and virus-related inflammatory pathways (Fig. 5E).

Venn diagram analysis of enriched pathways showed that TB-327 and TG-327 groups shared 122 common KEGG pathways, while 36 and 58 pathways were uniquely enriched in TB-327 and TG-327, respectively (Fig. 5F). Further classification of significantly enriched pathways revealed striking differences between TB-327 and TG-327 (Fig. S5). TB-327-specific pathways involved glycosaminoglycan biosynthesis, cardiac contraction, steroid biosynthesis, and NF-κB signaling, suggesting a bias toward structural and metabolic modulation. TG-327-specific pathways were dominated by immune and infection-related processes, such as systemic lupus erythematosus, viral infection, apoptosis, and p53 signaling, indicating a robust inflammatory and antiviral transcriptional program. The shared pathway subset was mainly composed of MAPK signaling, cancer pathways, and protein export, pointing to common but foundational oncogenic signals.

PCA demonstrated a clear separation between TB-327 and TG-327 groups transcriptomes (Fig. 5G). Samples from the two groups clustered tightly within their respective groups but were completely separated along PC1, which explained 86.89% of the total variance. This indicates strong intra-group consistency and profound inter-group transcriptomic divergence. Collectively, these findings demonstrate that TB-327 and TG-327 groups elicit distinct transcriptomic landscapes, characterized by non-overlapping DEGs, group-specific enriched pathways, and divergent biological functions. While TB-327 group is associated with metabolic and structural regulation, TG-327 group is associated with a stronger immunological and pathological response, underscoring their mechanistic divergence despite shared core pathway activations.

### Comparative transcriptomic analysis reveals cell-type-specific responses to BHI- cultured *S. hominis* supernatants

**Figure 6:**
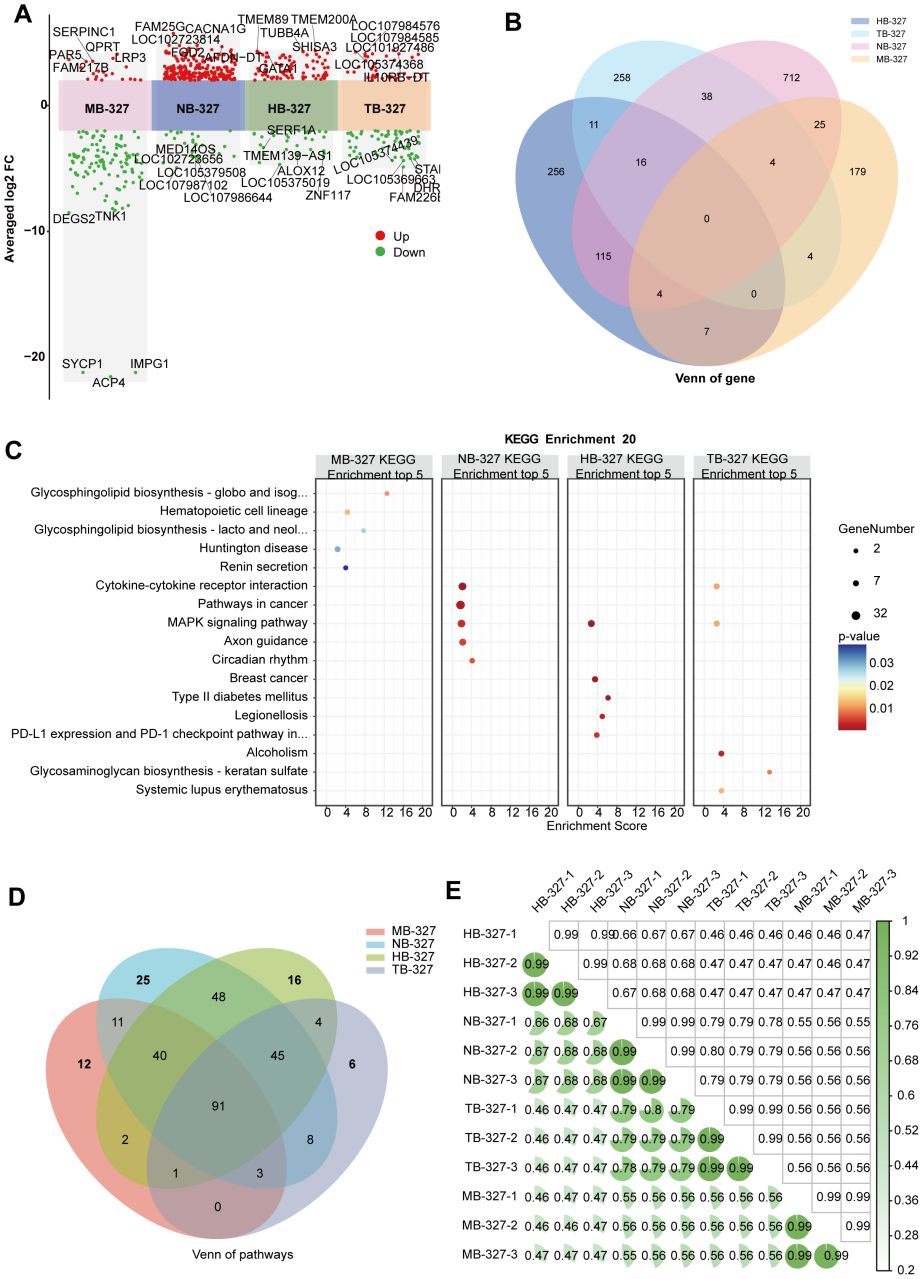
Transcriptomic comparative analysis of the responses of four cell lines to the supernatant of *S. hominis* cultured in BHI medium. (A) Volcano plots of DEGs in the MB-327, NB-327, HB-327, TB-327, *n* = 3 in per group. Red dots indicate significantly upregulated genes, green dots indicate significantly downregulated genes. Key DEGs are annotated. (B) Venn diagram showing overlapping and unique DEGs between the MB-327, NB-327, HB-327 and TB-327 groups compared to the NC group. The heatmaps display the expression levels of the top 10 upregulated and downregulated genes specific to MB-327, NB-327, HB-327 and TB-327. (C) KEGG pathway enrichment analysis of the top 5 significantly enriched pathways in the MB-327, NB-327, HB-327, and TB-327 group. (D) Venn diagram showing overlapping and unique KEGG-enriched pathways between the MB-327, NB-327, HB-327, and TB-327groups compared to the NC group. (E) Correlation heatmap showing the relationship between samples from the MB-327, NB-327, HB-327, and TB-327 groups based on gene expression, with three replicates per group. Differentially expressed genes were identified using DESeq2 with |log_2_FC| > 2 and *p* < 0.05 as the significance threshold.

To investigate the transcriptional responses of intestinal and non-intestinal epithelial cells to BHI-cultured *S. hominis* supernatant, RNA-seq was performed on MODE-K, NCM460, Henle-407, and HEK-293T cells. Mouse genes were mapped to their human orthologs *via* ENSEMBL. Multi-cluster fold change plots revealed distinct patterns of significantly upregulated (red) and downregulated (green) DEGs across the four cell types (—log2FC— > 2, *p* < 0.05) (Fig. 6A), underscoring the potency and cell type specificity of *S. hominis*-was associated with transcriptomic alterations. To quantify DEG similarity across cell types, Venn analysis of significantly regulated DEGs revealed minimal overlap among MB-327, NB-327, HB-327, and TB-327 groups, with most DEGs being unique to each (Fig. 6B). Only a few shared genes were identified, highlighting strong cell-type specificity in response to *S. hominis*. Heatmap-based hierarchical clustering further confirmed distinct gene expression signatures across groups (Fig. S6A).

KEGG enrichment analysis, based on significantly regulated DEGs (*p* < 0.05, Benjamini–Hochberg correction), revealed distinct cellular responses across the four cell types (Fig. 6C). The MB-327 group was enriched in pathways such as glycosphingolipid biosynthesis, hematopoietic cell lineage, and MAPK signaling. The NB-327 group showed enrichment in immune- and signaling-related pathways, including PD-L1/PD-1 checkpoint, cytokine–cytokine receptor interaction, and circadian rhythm. In the HB-327 group, enriched pathways were associated with renin secretion, alcoholism, and type II diabetes. The TB-327 group exhibited enrichment in glycosaminoglycan biosynthesis and keratan sulfate metabolism. These findings underscore the cell type-dependent functional effects of *S. hominis* supernatant.

To further dissect shared and unique responses, we compared all enriched KEGG pathways across the four cell types. Venn analysis revealed 91 pathways commonly enriched among all groups, indicating a conserved core response to BHI-cultured *S. hominis* supernatants (Fig. 6D). In contrast, each group also exhibited distinct pathway subsets-25 in MB-327group, 16 in NB-327 group, 12 in HB-327 group, and six in TB-327 group-highlighting cell type-specific functional divergence. To further understand the cell-specific transcriptional programs, we classified the unique pathways enriched in each group (Fig. S6B). TB-327 group-specific pathways included mucin-type O-glycan biosynthesis, cytosolic DNA sensing, and other glycan-related processes, indicating potential engagement of host-microbe interaction and mucosal barrier components. HB-327 group-specific responses involved amino acid metabolism, oxidative defense (peroxisome), and endocrine-related pathways such as thyroid cancer and selenocompound metabolism. NB-327 group uniquely enriched pathways related to digestion and immune sensing, such as protein digestion and virion response. MB-327 group was characterized by unique enrichment in Hippo signaling, branched-chain amino acid degradation, and other carbohydrate metabolic pathways. This diversity reflects different biological priorities and susceptibilities among cell types in response to the same microbial stimulus.

To evaluate sample consistency and overall transcriptional relationships, we conducted pairwise Pearson correlation analysis across all samples (Fig. 6E). Intra-group samples showed near-perfect correlation (*R* > 0.99), indicating high reproducibility and minimal technical noise. In contrast, inter-group correlations were significantly lower (generally *R* < 0.5), reinforcing the conclusion that each cell type responded uniquely to the same BHI-cultured *S. hominis* supernatants. Together, these results support the conclusion that host cell type is a dominant determinant of response to BHI-cultured *S. hominis* supernatants.

### Comparative transcriptomic analysis reveals cell-type-specific responses to GAM- cultured *S. hominis* supernatants

**Figure 7:**
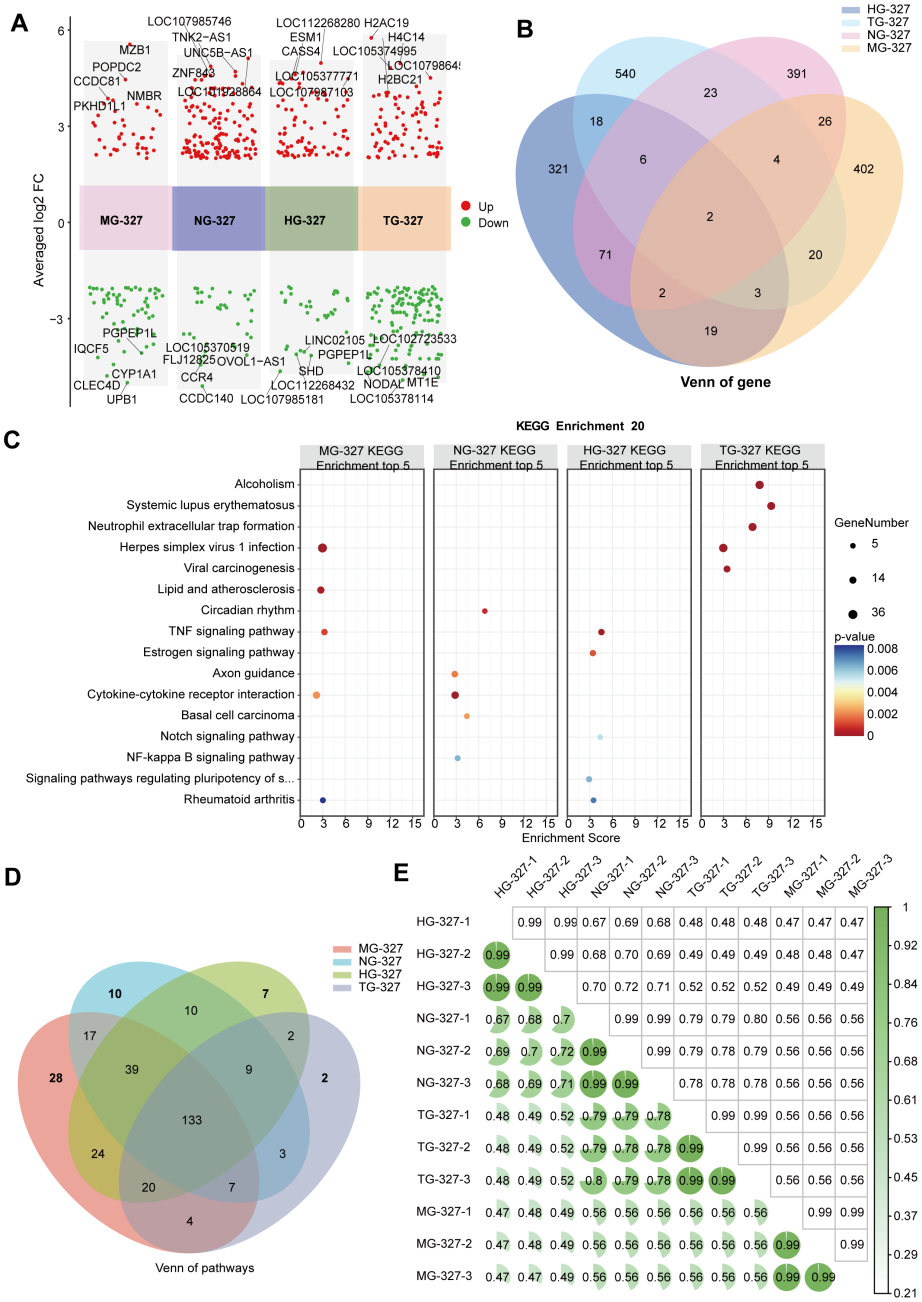
Transcriptomic comparative analysis of the responses of four cell lines to the supernatant of *S. hominis* cultured in GAM medium. (A) Volcano plots of DEGs in the MG-327, NG-327, HG-327 and TG-327, *n* = 3 in per group. Red dots indicate significantly upregulated genes, green dots indicate significantly downregulated genes. Key DEGs are annotated. (B) Venn diagram showing overlapping and unique DEGs between the MG-327, NG-327, HG-327 and TG-327 groups compared to the NC group. The heatmaps display the expression levels of the top 10 upregulated and downregulated genes specific to MG-327, NG-327, HG-327 and TG-327. (C) KEGG pathway enrichment analysis of the top 5 significantly enriched pathways in the MG-327, NG-327, HG-327 and TG-327 groups. (D) Venn diagram showing overlapping and unique KEGG-enriched pathways between the MG-327, NG-327, HG-327 and TG-327 groups compared to the NC group. (E) Correlation heatmap showing the relationship between samples from the MG-327, NG-327, HG-327 and TG-327 groups based on gene expression, with three replicates per group. Differentially expressed genes were identified using DESeq2 with |log_2_FC| > 2 and *p* < 0.05 as the significance threshold.

To further evaluate the universality and cell type specificity of the observed responses, we extended the transcriptome analysis to four epithelial cell models treated under GAM-cultured *S. hominis* supernatants. Multi-cluster fold change plots demonstrated that each cell type exhibited a distinct transcriptional response to *S. hominis* supernatant, with substantial numbers of significantly upregulated (red) and downregulated (green) DEGs (—log2FC— > 2, *p* < 0.05) (Fig. 7A), highlighting strong cell type-dependent effects. Venn analysis revealed extensive divergence in DEGs across cell lines, with MG-327, NG-327, HG-327, and TG-327 groups each containing over 100 unique genes. No genes were commonly regulated across four group (Fig. 7B), confirming the strong influence of cell identity on transcriptomic responses. Heatmap confirms group-specific gene expression signatures. Unsupervised hierarchical clustering of representative DEGs further supported the divergence of transcriptional profiles (Fig. S7A).

KEGG enrichment analysis, based on significantly regulated DEGs (*p* < 0.05, Benjamini–Hochberg correction), identified cell-specific functional programs. MG-327 group enriched in immune and cancer-related pathways; NG-327 group in hormonal and circadian pathways; HG-327 group in inflammatory signaling (*e.g.*, NF-κB, TNF); and TG-327 group in developmental pathways (Fig. 7C). These findings reflect diverse cellular adaptations to *S. hominis* exposure.

Despite 133 KEGG pathways shared across groups, each cell line also exhibited unique enrichments, such as glycan metabolism in NG-327, transcriptional regulation in MG-327, and endocrine signaling in HG-327 (Fig. 7D). This underscores a balance between conserved and cell-type-specific responses. Functional classification highlights divergent biological roles. Pathway categorization showed that MG-327 group responded *via* transcription and carbohydrate metabolism; NG-327 group *via* lipid and glycan processing; HG-327 group *via* amino acid metabolism and endocrine regulation; and TG-327 group *via* protein export and stress response (Fig. S7B), further confirming context-dependent functional divergence.

Correlation analysis validates sample consistency and inter-group diversity. Intra-group replicates exhibited high correlation (*R* > 0.99), while inter-group comparisons showed clear separation (*R* < 0.5) (Fig. 7E), confirming both experimental reproducibility and strong biological divergence across cell types. These results highlight the importance of host cell identity in shaping microbial response outcomes.

### Functional verification of inflammatory and metabolic responses

**Figure 8:**
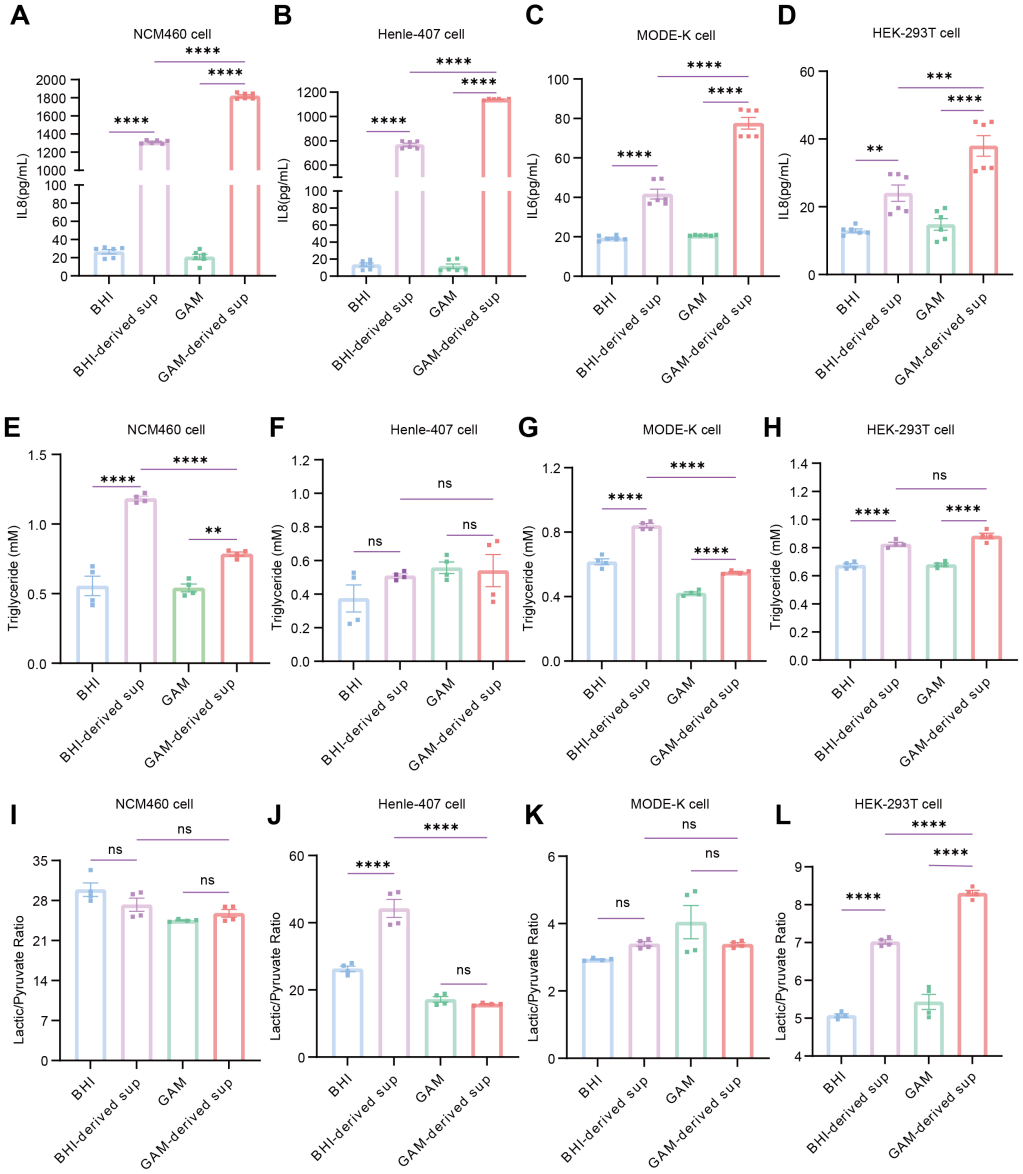
Functional validation of inflammatory and metabolic responses across cell lines. (A, B, D) IL-8 protein levels in NCM460, Henle-407, and HEK-293T cells following treatment with BHI-derived or GAM-derived *S. hominis* supernatants for 24 h, compared with medium-matched controls. (C) IL-6 protein levels in MODE-K cells treated under the same conditions. Data represent mean ± SEM (*n* = 2 biological replicates × 3 technical replicates per condition). Statistical analysis was performed using one-way ANOVA with Tukey’s multiple-comparison test (*p* < 0.05; ** *p* < 0.01; *** *p* < 0.001; **** *p* < 0.0001; ns, not significant). (E–H) Intracellular triglyceride content in NCM460, MODE-K, Henle-407, and HEK-293T cells following 24 h exposure to the indicated supernatants. (I–M) Lactate/pyruvate (L/P) ratio measured in the same cell lines after 6 h treatment. Data represent mean ± SEM (*n* = 2 biological replicates × 2 technical replicates per condition). Statistical analysis was performed using one-way ANOVA with Tukey’s multiple-comparison test (*p* < 0.05; ** *p* < 0.01; *** *p* < 0.001; **** *p* < 0.0001; ns, not significant).

To verify the key response patterns suggested by the transcriptomic analysis, we performed targeted functional assays encompassing cytokine secretion and metabolic indicators across the four cell lines. When exposed to GAM-derived *S. hominis* supernatants, IL-8 protein levels were significantly increased in NCM460, Henle-407, and HEK-293T cells relative to untreated and medium-matched controls, as well as BHI-derived supernatants (*p* < 0.0001) (Figs. 8A–8C). In MODE-K cells, where IL-6 represented the predominant cytokine response, IL-6 secretion was similarly elevated under GAM treatment (*p* < 0.0001) (Fig. 8D). These observations suggest that bacterial products generated under GAM conditions may exert a comparatively greater influence on epithelial inflammatory signaling.

We next assessed metabolic phenotypes associated with BHI-derived *S. hominis* supernatants by measuring intracellular triglyceride levels and the lactate/pyruvate (L/P) ratio. Triglyceride content was increased in NCM460, MODE-K, and HEK-293T cells following BHI treatment (*p* < 0.0001), whereas no significant change was observed in Henle-407 cells (Figs. 8E–8H). In contrast, the L/P ratio was elevated in Henle-407 and HEK-293T cells exposed to BHI-derived *S. hominis* supernatants (*p* < 0.0001), while NCM460 and MODE-K cells did not show significant differences in this measurement (Figs. 8I–8M). These results indicate that BHI-conditioned *S. hominis* products are associated with alterations in cellular metabolic activity, with cell type-specific metabolic response patterns.

## Discussion

In this study, we combined bacterial metabolomics, multi-cell line transcriptomics, and functional assays to investigate how culture conditions and host cell type jointly shape epithelial responses to a gut-derived *S. hominis* isolate. Using supernatants from bacteria grown in either BHI or GAM medium to stimulate four models-MODE-K (mouse-derived), NCM460 and Henle-407 (both human-derived), and HEK-293T (a widely used epithelial “tool” cell line)-we found that: (1) the bacterial nutritional environment drives pronounced remodeling of the *S. hominis* exometabolome, (2) host responses to microbiota cultured under varying conditions within a consistent host background, and (3) host-specific responses elicited by identically cultured microbiota across different cell types. Together, these data argue that “microbial context” (culture medium) and “host context” (cell type) are inseparable variables *in vitro* microbiota-host interaction studies.

Rigorous quality control procedures confirmed the robustness of reliability of both the metabolomic and the transcriptomic data. The identified DMGs captured metabolic differences in *S. hominis* between the two culture conditions, whereas the DEGs reflected host cellular responses to exposure to *S. hominis* supernatants. The dataset demonstrated excellent reproducibility and biological significance, supporting their suitability for downstream functional analyses.

One of the main observations in this study is that *S. hominis* displays distinct extracellular metabolite profiles under BHI and GAM culture conditions. Untargeted metabolomics revealed extensive medium-dependent remodeling, with both shared and condition-specific differential metabolites. This is consistent with previous work showing that growth medium and environmental parameters can profoundly alter bacterial traits, including virulence/regulatory gene expression in Staphylococcus aureus (Ray, Ballal & Manna, 2009), metabolite profiles and host gene expression in response to changes in pH, CO_2_, and nutrients (Chan & Foster, 1998), and broader metabolic characteristics under different culture conditions (Mielko et al., 2019). In our dataset, fatty acyls and glycerophospholipids were mainly linked to BHI-derived *S. hominis* supernatants and to pathways of carbohydrate/energy metabolism and selected amino acid/lipid metabolism, in line with reported roles of these lipid classes in membrane structure and cellular metabolic regulation (Tremaroli & Bäckhed, 2012; Van Meer, Voelker & Feigenson, 2008). In contrast, sphingolipids and organic carbonic acids and their derivatives were more abundant under GAM conditions, with mainly enrichment in purine, α-linolenic acid, and arachidonic acid metabolism, lipid axes that have been repeatedly linked to inflammatory signaling and immune modulation (Calder, 2015; Hannun & Obeid, 2018). These findings suggest that the extracellular metabolite composition of *S. hominis* is sensitive to the nutritional environment and may modulate the balance of metabolite combinations associated with metabolic *versus* inflammation-related host responses.

Across MODE-K, NCM460, Henle-407, or HEK-293T cells, *S. hominis* supernatants derived from BHI and GAM media were associated with distinct transcriptional responses, as reflected by differences in the number of DEGs, limited gene overlap, and divergent KEGG pathway enrichments. These observations suggest that bacterial culture conditions play a critical role in modulating host cell responses.

BHI-derived supernatants in our system were more often linked to metabolic, membrane/extracellular matrix, and growth-related pathways, whereas GAM-derived supernatants were more often linked to immune and inflammatory pathways, In MODE-K and NCM460 cells, BHI-derived supernatants preferentially enriched pathways related to lipid handling, extracellular matrix (ECM) organization, and proliferative signaling. In HEK-293T cells, TB-327 was associated with mucin-type O-glycan and keratan sulfate glycosaminoglycan biosynthesis together with MAPK and TGF-β signaling, while in Henle-407 cells HB-327 showed enrichment of PI3K–Akt and MAPK signaling, natural killer cell–mediated cytotoxicity.

In contrast, GAM-derived supernatants were associated with stronger enrichment of immune-, infection- and cell-death-related pathways across the four cell lines. In MODE-K and NCM460 cells, this included enrichment of IL-17, TNF and NF-κB signaling, cytokine-cytokine receptor interaction, cytosolic DNA-sensing, viral interaction pathways, and infection-related modules. In HEK-293T cells, TG-327 was linked to MAPK and neutrophil extracellular trap formation, whereas in Henle-407 cells HG-327 showed enrichment of sphingolipid and Notch signaling. These results suggest that BHI-grown bacteria are more closely associated with metabolic and matrix-remodeling programs, whereas GAM-grown bacteria more often engage inflammation- and infection-related pathways, with each cell line superimposing its own regulatory preferences on this overall pattern. This is consistent with previous reports that gut microbes and their metabolites can modulate both host metabolism and immunity. These findings align with previous transcriptomic studies showing that culture medium composition significantly influences bacterial functional outputs. For example, *Pseudomonas* species display medium-dependent variation in protein synthesis, energy metabolism, and secondary metabolite production (Yang et al., 2016), while *Bacteroides uniformis* CECT 7771 alters its transcriptional activity and glycolytic capacity depending on available carbon sources (Benítez-Páez, Gómez Del Pulgar & Sanz, 2017). Our results extend these insights by showing that such microbial plasticity also dictates distinct transcriptional landscapes in host cells, thereby highlighting the influence of microbial growth conditions in host-microbe interactions. These data emphasize the necessity of accounting for microbial culture conditions when designing and interpreting *in vitro* microbiota-host interaction models.

At the same time, our data highlight that host cell identity is a major determinant of how a given *S. hominis* supernatant is interpreted. Even when exposed to the same *S. hominis* supernatants, MODE-K, NCM460, Henle-407, and HEK-293T cells showed minimal overlap at the individual DEG level, with most genes being unique to each cell type. KEGG analysis revealed a conserved core of shared pathways but also a substantial number of cell type-specific enrichments. For example, in MODE-K cells, MB-327 group enrichment in glycosphingolipid biosynthesis (globo/isoglobo and lacto/neolacto series). These observations are in line with prior findings that the microbial metabolite indole-3-propionic acid can prevent radiation toxicity by stabilizing acyl-CoA-binding proteins in intestinal epithelial cells, highlighting the metabolic adaptability of MODE-K cells in host-microbe interactions (Xiao et al., 2020). Similarly, NB-327 induced transcriptomic signatures in NCM460 cells that were enriched in secondary metabolite biosynthesis, lipid metabolism, and cell signaling pathways. This is consistent with earlier studies using NCM460 to investigate lipid metabolism disruptions caused by aflatoxins B1 and M1 (Yang et al., 2023), and butyrate-mediated regulation of host signaling (Zeng et al., 2017), reinforcing their suitability for metabolic and immunological studies. In Henle-407 cells, HB-327-specific enrichments involved pathways linked to endocrine function, oxidative stress, and immune responses-findings that align with their common use in investigating enteric pathogen infection, such as *Salmonella* (Sun et al., 2018). HEK-293T cells, though non-intestinal, also exhibited distinct transcriptional features in response to TB-327, including enrichment in glycan biosynthesis, cytosolic DNA sensing, and antiviral defense pathways. These results correspond with previous functional metagenomic studies demonstrating that bacterial effectors can modulate gene networks and antiviral defenses in HEK-293T cells (Cohen et al., 2015). Similarly, in GAM medium, the four host cell types also exhibit comparable responses. Strobel et al. (2016) also highlighted the specific impacts of different strains and cell types on infection mechanisms and supported an in-depth understanding of strain-host cell interactions through proteomics data. Prior research has emphasized that cellular identity strongly influences responsiveness to microbial cues, including toll-like receptor expression, interferon competence, and mucosal defense programs (Belkaid & Harrison, 2017). Interestingly, while each cell line responded uniquely, a conserved set of core immune pathways-such as MAPK, NF-κB, and cytokine-cytokine receptor interactions-were recurrent across conditions. This suggests a shared baseline capacity among epithelial cells to sense and respond to microbial factors, but with significant tuning depending on microbial environment and host cellular context. Together, these findings suggest that the same microbial products can elicit distinct, cell type-specific response patterns because they are interpreted through different receptor repertoires, signaling networks, and baseline transcriptional states. This is consistent with previous multi-cell line studies showing that cell identity strongly shapes stimulus-induced transcriptional programs (Cursons et al., 2015) and underscores intrinsic differences in epithelial physiology and susceptibility.

In addition to intestinal models, we included HEK-293T cells, a non-intestinal epithelial line with defined genetics and low basal activity widely used to study innate immune and NF-κB signaling (Duncan et al., 2017). HEK-293T provides a simplified and genetically stable background compared with heterogeneous intestinal cell lines, thereby facilitating the identification of fundamental and broadly conserved transcriptional responses to *S. hominis*-derived stimuli under different culture conditions. In our study, HEK-293T cells shared conserved MAPK and NF-κB programs with intestinal models but also showed tissue-specific enrichment (*e.g.*, glycosaminoglycan pathways in BHI, antiviral and cell death pathways in GAM), highlighting context-dependent sensitivity to microbial stimuli.

Importantly, the functional assays were broadly consistent with the patterns suggested by the omics analyses and helped to move beyond purely transcriptional correlations. Cytokine ELISAs showed that GAM-derived *S. hominis* supernatants were associated with higher IL-8 secretion in NCM460, Henle-407, and HEK-293T cells, and higher IL-6 secretion in MODE-K cells, compared with untreated and medium controls as well as BHI-derived supernatants. This cytokine profile is in line with the transcriptomic enrichment of TNF, IL-17 and related inflammation-associated pathways under GAM conditions, and with the established roles of these signaling axes in driving epithelial inflammatory responses (Bradford et al., 2017; McGeachy, Cua & Gaffen, 2019) Conversely, BHI-derived supernatants were more closely linked to metabolic readouts: intracellular triglyceride levels were increased in NCM460, MODE-K, and HEK-293T cells, a parameter widely used to reflect cellular lipid storage and metabolic reprogramming (Listenberger et al., 2003; Samuel & Shulman, 2012), while the lactate/pyruvate ratio-commonly used as a proxy for the cytosolic NADH/NAD^+^ redox state (Go et al., 2021; Lin, Wang & Li, 2022) was elevated in Henle-407 and HEK-293T cells. These changes are consistent with the enrichment of lipid metabolism, glycerophospholipid metabolism, and energy metabolism pathways under BHI conditions, and suggest that *S. hominis* products generated in nutrient-rich media may modulate lipid storage and redox-related parameters in a cell type-dependent manner. Notably, the metabolic and inflammatory responses did not fully overlap across models (for example, Henle-407 cells did not show triglyceride accumulation but did display an altered lactate/pyruvate ratio), indicating that different epithelial cell types emphasize distinct facets of the microbial stimulus.

This study underscores that host-microbe interaction profiles are highly sensitive to experimental context, including bacterial growth conditions and host cell type. Most prior *in vitro* studies use standard culture media and generic cell lines, overlooking the complexity of host tissue identity and microbial environmental adaptation. Our findings align with recent calls for more personalized or context-aware microbiome research, particularly in therapeutic microbiome modulation strategies (Gilbert et al., 2025).

However, there are limitations to this work. Although metabolomic and transcriptomic analyses provides insights into global and system-level patterns, they remain correlative and cannot distinguish direct actions of specific bacterial products from secondary host signaling cascades. Targeted perturbation of candidate metabolites, microbial pathways, or host receptors will be required to establish causality. Moreover, functional validation was restricted to a limited set of readouts (IL-6/IL-8 secretion, triglycerides, lactate/pyruvate ratio), and other key epithelial functions (barrier integrity, cell death, differentiation, and crosstalk with immune cells) were not examined and warrant further investigation. In addition, the use of immortalized cell lines, while convenient, may not fully recapitulate *in vivo* epithelial physiology or host-microbe interface dynamics. Primary cells, organoids, or *in vivo* models could help extend the findings.

In summary, our study demonstrates that the functional outcomes of *S. hominis* exposure are co-determined by bacterial culture environment and host cell identity. In our models, variation in culture medium and cell type was accompanied by systematic changes in the exometabolite profile, host transcriptional programs, and downstream inflammatory and metabolic readouts. These observations indicate that neither the microbial “input” nor the host “background” is a neutral parameter *in vitro*, and that both should be taken into account when designing and interpreting microbiota–host interaction experiments. More broadly, this context dependence may have implications for microbiome-based diagnostics and interventions.

## Conclusion

In conclusion, this study suggests that epithelial responses to a gut-derived *S. hominis* isolate are influenced by both the bacterial growth environment and the identity of the host cell. By combining metabolomic profiling of BHI- and GAM-derived supernatants with multi-cell line transcriptomics and targeted functional assays, we observed that medium-dependent remodeling of the *S. hominis* exometabolome was accompanied by a shift in host response profiles, with BHI-derived supernatants more often associated with metabolic and structural pathways, and GAM-derived supernatants more frequently linked to immune- and inflammation-related signaling. At the same time, transcriptional and functional readouts were largely cell type–specific: MODE-K, NCM460, Henle-407, and HEK-293T cells each exhibited distinct sets of DEGs, pathway enrichments, cytokine patterns, and metabolic changes in response to the same bacterial stimuli, while still sharing a conserved core of immune signaling pathways. These observations indicate that neither the microbial “input” (culture conditions and exometabolite composition) nor the host “background” (cell type) should be regarded as neutral parameters *in vitro*, and that both factors merit explicit consideration when designing and interpreting microbiota–host interaction experiments. More broadly, the framework used here to connect culture-dependent exometabolomes with cell-specific transcriptional and functional responses may be useful for developing more context-aware *in vitro* models and for informing future tissue-targeted microbiome-based diagnostic and therapeutic strategies.

##  Supplemental Information

10.7717/peerj.20899/supp-1 Supplemental Information 1 Sequencing data statistics Sequencing data statistics. Note: The following naming conventions are used for the cells treated with BHI medium, MODEK cells treated with BHI medium are named MB-NC; MODE-K cells treated with * S. hominis* metabolites cultured in BHI medium are named MB-327; NCM460 cells treated with BHI medium are named NB-NC; NCM460 cells treated with * S. hominis* supernatant cultured in BHI medium are named NB-327; Henle- 407 cells treated with BHI medium are named HB-NC; Henle-407 cells treated with * S. hominis* supernatant cultured in BHI medium are named HB-327; HEK-293T cells treated with BHI medium are named TB-NC; HEK-293T cells treated with * S. hominis* supernatant cultured in BHI medium are named TB-327. Each group has three biological replicates.

10.7717/peerj.20899/supp-2 Supplemental Information 2 Sequencing data statistics Sequencing data statistics. Note: The following naming conventions are used for the cells treated with GAM medium, MODE-K cells treated with GAM medium are named MG-NC; MODE-K cells treated with * S. hominis* supernatant cultured in GAM medium are named MG-327; NCM460 cells treated with GAM medium are named NG-NC; NCM460 cells treated with * S. hominis* supernatant cultured in GAM medium are named NG-327; Henle-407 cells treated with GAM medium are named HG-NC; Henle-407 cells treated with * S. hominis* supernatant cultured in GAM medium are named HG-327; HEK-293T cells treated with GAM medium are named TG-NC; HEK-293T cells treated with * S. hominis* supernatant cultured in GAM medium are named TG-327. Each group has three biological replicates.

10.7717/peerj.20899/supp-3 Supplemental Information 3 Genome circle plot, QC sample correlation analysis and number of genes detected in each sample (A) Basic genomic information of *S. hominis*. B Schematic representation of the experimental design and workflow for metabolomic and transcriptomic analyses. (C–D) Pairwise Pearson correlation matrices of QC samples in negative and positive ion modes. Correlation coefficients close to 1 indicate high analytical reproducibility and stable instrument performance. (E–F) Total number of genes identified in MODE-K, Henle-407, NCM460, and HEK-293T cell lines through transcriptomic analyses.

10.7717/peerj.20899/supp-4 Supplemental Information 4 Transcriptomic changes in MODE-K cell line KEGG pathway enrichment analysis the overlapping and unique top 10 significantly enriched pathways in the MB-327 and MG-327 group, each compared to the NC group.

10.7717/peerj.20899/supp-5 Supplemental Information 5 Transcriptomic changes in NCM460 cell line KEGG pathway enrichment analysis the overlapping and unique top 10 significantly enriched pathways in the NB-327 and NG-327, each compared to the NC group.

10.7717/peerj.20899/supp-6 Supplemental Information 6 Transcriptomic changes in Helen-407 cell line KEGG pathway enrichment analysis the overlapping and unique top 10 significantly enriched pathways in the HB-327 and HG-327, each compared to the NC group.

10.7717/peerj.20899/supp-7 Supplemental Information 7 Transcriptomic changes in HEK-293T cell line KEGG pathway enrichment analysis the overlapping and unique top 10 significantly enriched pathways in the TB-327 and TG-327, each compared to the NC group.

10.7717/peerj.20899/supp-8 Supplemental Information 8 Transcriptomic comparative analysis of the responses of four cell lines to the supernatant of *S. hominis* cultured in BHI medium (A) Heatmap showing the expression of significantly differentially expressed genes in MB-327, NB-327, HB-327, and TB-327, with red indicating upregulated genes and blue indicating downregulated genes. (B) KEGG pathway enrichment analysis the unique top 6 significantly enriched pathways in the MB-327, NB-327, HB-327 and TB-327, each compared to the NC group.

10.7717/peerj.20899/supp-9 Supplemental Information 9 Transcriptomic comparative analysis of the responses of four cell lines to the supernatant of *S. hominis* cultured in GAM medium (A) Heatmap showing the expression of significantly differentially expressed genes in MG-327, NG-327, HG-327 and TG-327, with red indicating upregulated genes and blue indicating downregulated genes. (B) KEGG pathway enrichment analysis the unique top 5 significantly enriched pathways in the MG-327, NG-327, HG-327 and TG-327 groups, each compared to the NC group.

## Abbreviations

BHI: Brain-heart infusion
GAM: Gifu Anaerobic Medium
SCFAs: Short-chain fatty acids
BMDMs: Marrow-derived macrophages
DEG: Differentially expressed gene
KEGG: Kyoto Encyclopedia of Genes and Genomes
PCA: Principal component analysis
DMEM: Dulbecco’s Eagle Medium, High Glucose
RNA: RNA-Seqtranscriptome sequencing

## Additional Information and Declarations

### Data Availability

The following information was supplied regarding data availability: The datasets generated in this study are available in the NCBI Sequence Read Archive (SRA): PRJNA1226736 and PRJNA1226357. The metabolomic data are available in the GSA-OMIX database: OMIX013095.
