# Supplementary material for: Transcriptomic and metabolomic profiling reveals media- and host-dependent responses to Staphylococcus hominis in cell models
Source: PeerJ. 2026 Mar 12;14:e20899. doi: 10.7717/peerj.20899 (PMC12989153; doi:10.7717/peerj.20899)
Supplement: Supplemental Information 1 — Sequencing data statistics. Note: The following naming conventions are used for the cells treated with BHI medium, MODEK cells treated with BHI medium are named MB-NC; MODE-K cells treated with S. hominis metabolites cultured in BHI medium are named MB-327; NCM460 cells treated with BHI medium are named NB-NC; NCM460 cells treated with S. hominis supernatant cultured in BHI medium are named NB-327; Henle- 407 cells treated with BHI medium are named HB-NC; Henle-407 cells treated with S. hominis supernatant cultured in BHI medium are named HB-327; HEK-293T cells treated with BHI medium are named TB-NC; HEK-293T cells treated with S. hominis supernatant cultured in BHI medium are named TB-327. Each group has three biological replicates. [file peerj-14-20899-s001.docx]

Supplementary table 1 Sequencing data statistics

| Sample | RawReads  (M) | RawBases  (G) | CleanReads  (M) | CleanBases  (G) | ValidBases  (%) | Q30  (%) | GC  (%) |
| --- | --- | --- | --- | --- | --- | --- | --- |
| MB-NC-1 | 51.16 | 7.35 | 48.9 | 7.03 | 95.58 | 96.46 | 48.4 |
| MB-NC-2 | 49.68 | 7.22 | 48.01 | 6.98 | 96.64 | 95.76 | 50.6 |
| MB-NC-3 | 50.09 | 7.29 | 48.46 | 7.05 | 96.75 | 95.95 | 50.72 |
| HB-NC-1 | 50.71 | 7.27 | 48.3 | 6.92 | 95.25 | 97.32 | 49.76 |
| HB-NC-2 | 50.77 | 7.29 | 48.47 | 6.96 | 95.46 | 97.3 | 49.84 |
| HB-NC-3 | 52.13 | 7.44 | 49.41 | 7.05 | 94.79 | 97.26 | 49.86 |
| NB-NC-1 | 50.68 | 7.26 | 48.22 | 6.9 | 95.14 | 94.98 | 49.73 |
| NB-NC-2 | 50.53 | 7.24 | 48.09 | 6.89 | 95.17 | 96.25 | 49.85 |
| NB-NC-3 | 51.15 | 7.35 | 48.88 | 7.03 | 95.55 | 96.27 | 49.64 |
| TB-NC-1 | 52.03 | 7.38 | 48.99 | 6.95 | 94.17 | 96.09 | 50.26 |
| TB-NC-2 | 50.38 | 7.24 | 48.15 | 6.92 | 95.57 | 96.44 | 49.48 |
| TB-NC-3 | 52.29 | 7.45 | 49.48 | 7.05 | 94.62 | 95.13 | 49.06 |
| MB-327-1 | 49.76 | 7.19 | 47.67 | 6.89 | 95.8 | 96.09 | 50.93 |
| MB-327-2 | 48.55 | 7.11 | 47.3 | 6.93 | 97.42 | 96.4 | 50.54 |
| MB-327-3 | 49.47 | 7.19 | 47.83 | 6.95 | 96.68 | 94.66 | 50.81 |
| HB-327-1 | 52.46 | 7.42 | 49.28 | 6.97 | 93.93 | 97.43 | 51.17 |
| HB-327-2 | 52.91 | 7.47 | 49.53 | 6.99 | 93.6 | 97.36 | 50.98 |
| HB-327-3 | 51.94 | 7.4 | 49.17 | 7.01 | 94.66 | 97.37 | 50.2 |
| NB-327-1 | 52.2 | 7.44 | 49.37 | 7.03 | 94.58 | 96.36 | 50.06 |
| NB-327-2 | 51.09 | 7.28 | 48.3 | 6.88 | 94.55 | 96.32 | 51.14 |
| NB-327-3 | 51.48 | 7.31 | 48.59 | 6.9 | 94.38 | 95.92 | 50.71 |
| TB-327-1 | 51.77 | 7.35 | 48.81 | 6.93 | 94.28 | 95.14 | 49.87 |
| TB-327-2 | 51.55 | 7.33 | 48.68 | 6.92 | 94.43 | 96.26 | 49.55 |
| TB-327-3 | 51.23 | 7.32 | 48.65 | 6.95 | 94.98 | 96.39 | 49.23 |

Note: The following naming conventions are used for the cells treated with BHI medium, MODEK cells treated with BHI medium are named MB-NC; MODE-K cells treated with *S. hominis* metabolites cultured in BHI medium are named MB-327; NCM460 cells treated with BHI medium are named NB-NC; NCM460 cells treated with *S. hominis* supernatant cultured in BHI medium are named NB-327; Henle- 407 cells treated with BHI medium are named HB-NC; Henle-407 cells treated with *S. hominis* supernatant cultured in BHI medium are named HB-327; HEK-293T cells treated with BHI medium are named TB-NC; HEK-293T cells treated with *S. hominis* supernatant cultured in BHI medium are named TB-327. Each group has three biological replicates.
