# Supplementary material for: Transcriptomic and metabolomic profiling reveals media- and host-dependent responses to Staphylococcus hominis in cell models
Source: PeerJ. 2026 Mar 12;14:e20899. doi: 10.7717/peerj.20899 (PMC12989153; doi:10.7717/peerj.20899)
Supplement: Supplemental Information 2 — Sequencing data statistics. Note: The following naming conventions are used for the cells treated with GAM medium, MODE-K cells treated with GAM medium are named MG-NC; MODE-K cells treated with S. hominis supernatant cultured in GAM medium are named MG-327; NCM460 cells treated with GAM medium are named NG-NC; NCM460 cells treated with S. hominis supernatant cultured in GAM medium are named NG-327; Henle-407 cells treated with GAM medium are named HG-NC; Henle-407 cells treated with S. hominis supernatant cultured in GAM medium are named HG-327; HEK-293T cells treated with GAM medium are named TG-NC; HEK-293T cells treated with S. hominis supernatant cultured in GAM medium are named TG-327. Each group has three biological replicates. [file peerj-14-20899-s002.docx]

Supplementary table 2 Sequencing data statistics

| Sample | RawReads  (M) | RawBases  (G) | CleanReads  (M) | CleanBases  (G) | ValidBases  (%) | Q30  (%) | GC  (%) |
| --- | --- | --- | --- | --- | --- | --- | --- |
| MG-NC-1 | 49.46 | 7.22 | 47.99 | 7 | 97.04 | 96.42 | 50.64 |
| MG-NC-2 | 50.02 | 7.24 | 48.16 | 6.98 | 96.29 | 94.57 | 50.92 |
| MG-NC-3 | 49.13 | 7.14 | 47.49 | 6.9 | 96.65 | 96.01 | 50.85 |
| HG-NC-1 | 52.24 | 7.45 | 49.45 | 7.05 | 94.67 | 97.22 | 49.85 |
| HG-NC-2 | 50.89 | 7.28 | 48.35 | 6.91 | 95.01 | 97.23 | 49.76 |
| HG-NC-3 | 51.28 | 7.35 | 48.85 | 7 | 95.26 | 97.44 | 49.78 |
| NG-NC-1 | 50.59 | 7.29 | 48.48 | 6.99 | 95.82 | 96.2 | 49.85 |
| NG-NC-2 | 50.97 | 7.31 | 48.61 | 6.97 | 95.36 | 96.11 | 49.73 |
| NG-NC-3 | 52.07 | 7.43 | 49.32 | 7.03 | 94.72 | 96.23 | 50.6 |
| TG-NC-1 | 52 | 7.43 | 49.39 | 7.06 | 94.98 | 96.45 | 49.08 |
| TG-NC-2 | 51.74 | 7.35 | 48.81 | 6.93 | 94.34 | 96.56 | 50.12 |
| TG-NC-3 | 51.21 | 7.3 | 48.5 | 6.92 | 94.71 | 96.56 | 50.49 |
| MG-327-1 | 49.41 | 7.2 | 47.91 | 6.98 | 96.97 | 96.02 | 50.65 |
| MG-327-2 | 48.89 | 7.11 | 47.29 | 6.88 | 96.71 | 96.09 | 50.48 |
| MG-327-3 | 51.27 | 7.34 | 48.8 | 6.99 | 95.19 | 96.51 | 50.72 |
| HG-327-1 | 50.85 | 7.26 | 48.26 | 6.89 | 94.92 | 97.35 | 49.66 |
| HG-327-2 | 52.05 | 7.44 | 49.4 | 7.06 | 94.91 | 96.32 | 49.59 |
| HG-327-3 | 51.31 | 7.34 | 48.77 | 6.98 | 95.06 | 97.28 | 49.41 |
| NG-327-1 | 53.7 | 7.51 | 49.73 | 6.95 | 92.62 | 96.48 | 52.12 |
| NG-327-2 | 52.47 | 7.45 | 49.47 | 7.02 | 94.28 | 95.92 | 50.79 |
| NG-327-3 | 50.38 | 7.22 | 47.97 | 6.87 | 95.23 | 96.37 | 49.91 |
| TG-327-1 | 50.63 | 7.27 | 48.28 | 6.93 | 95.36 | 96.31 | 49.58 |
| TG-327-2 | 51.55 | 7.32 | 48.65 | 6.91 | 94.36 | 96.15 | 49.44 |
| TG-327-3 | 53.93 | 7.53 | 49.92 | 6.97 | 92.55 | 96.52 | 51.32 |

Note: The following naming conventions are used for the cells treated with GAM medium, MODE-K cells treated with GAM medium are named MG-NC; MODE-K cells treated with *S. hominis* supernatant cultured in GAM medium are named MG-327; NCM460 cells treated with GAM medium are named NG-NC; NCM460 cells treated with *S. hominis* supernatant cultured in GAM medium are named NG-327; Henle-407 cells treated with GAM medium are named HG-NC; Henle-407 cells treated with *S. hominis* supernatant cultured in GAM medium are named HG-327; HEK-293T cells treated with GAM medium are named TG-NC; HEK-293T cells treated with *S. hominis* supernatant cultured in GAM medium are named TG-327. Each group has three biological replicates.
