# Supplementary figures and images for: Transcriptomic and metabolomic profiling reveals media- and host-dependent responses to Staphylococcus hominis in cell models
Source: PeerJ. 2026 Mar 12;14:e20899. doi: 10.7717/peerj.20899 (PMC12989153; doi:10.7717/peerj.20899)

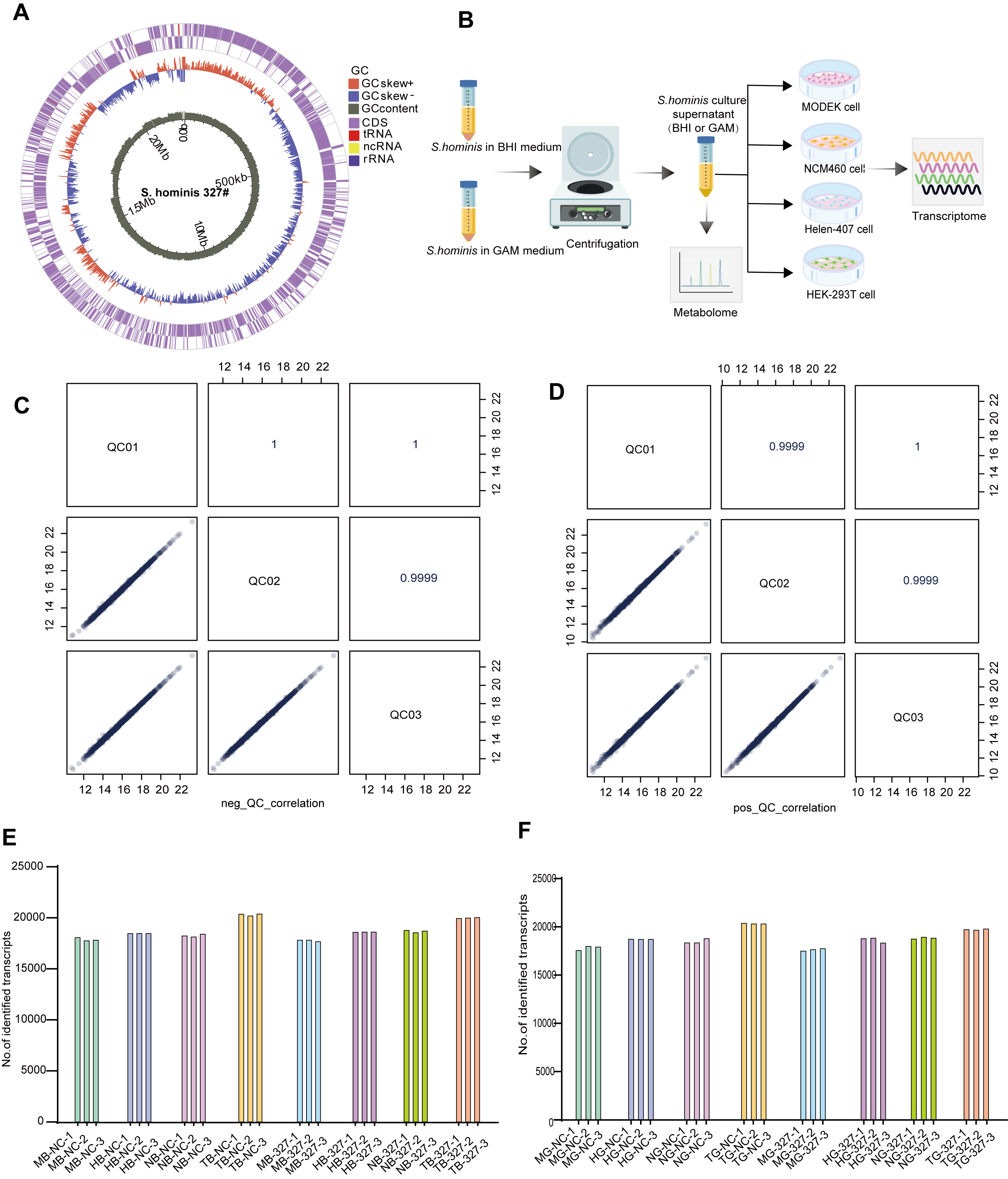

Supplement: Supplemental Information 3 — (A) Basic genomic information of S. hominis. B Schematic representation of the experimental design and workflow for metabolomic and transcriptomic analyses. (C–D) Pairwise Pearson correlation matrices of QC samples in negative and positive ion modes. Correlation coefficients close to 1 indicate high analytical reproducibility and stable instrument performance. (E–F) Total number of genes identified in MODE-K, Henle-407, NCM460, and HEK-293T cell lines through transcriptomic analyses. [file peerj-14-20899-s003.png]

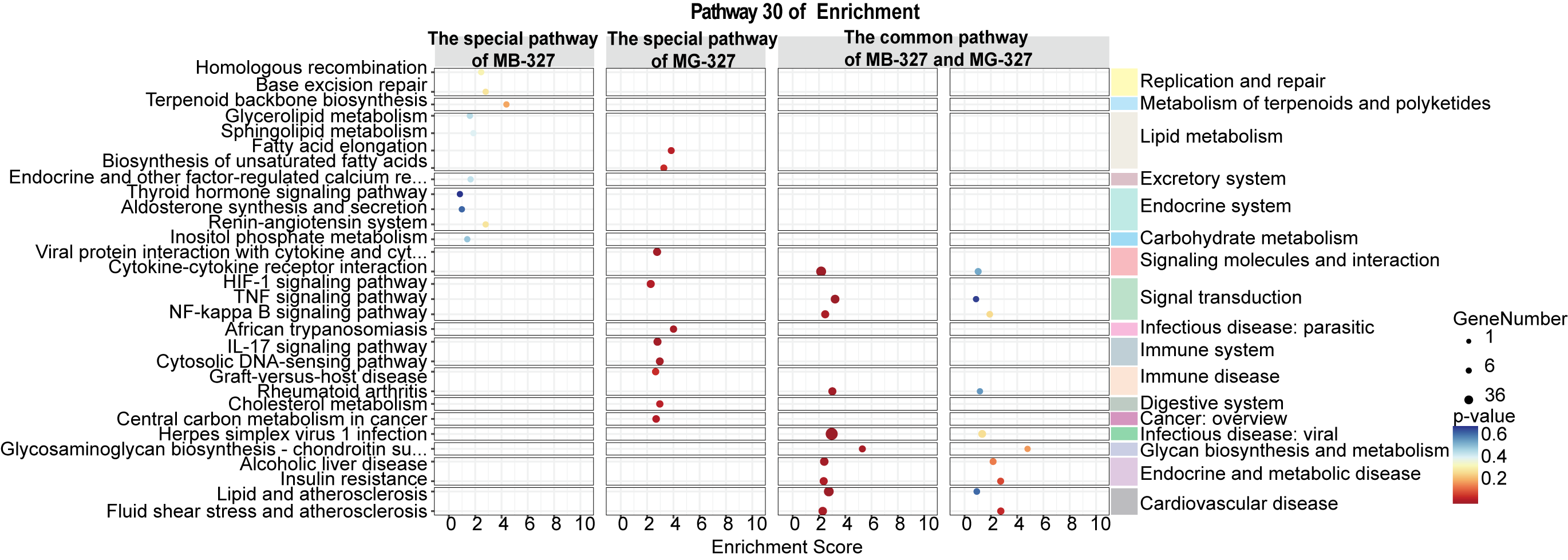

Supplement: Supplemental Information 4 — KEGG pathway enrichment analysis the overlapping and unique top 10 significantly enriched pathways in the MB-327 and MG-327 group, each compared to the NC group. [file peerj-14-20899-s004.png]

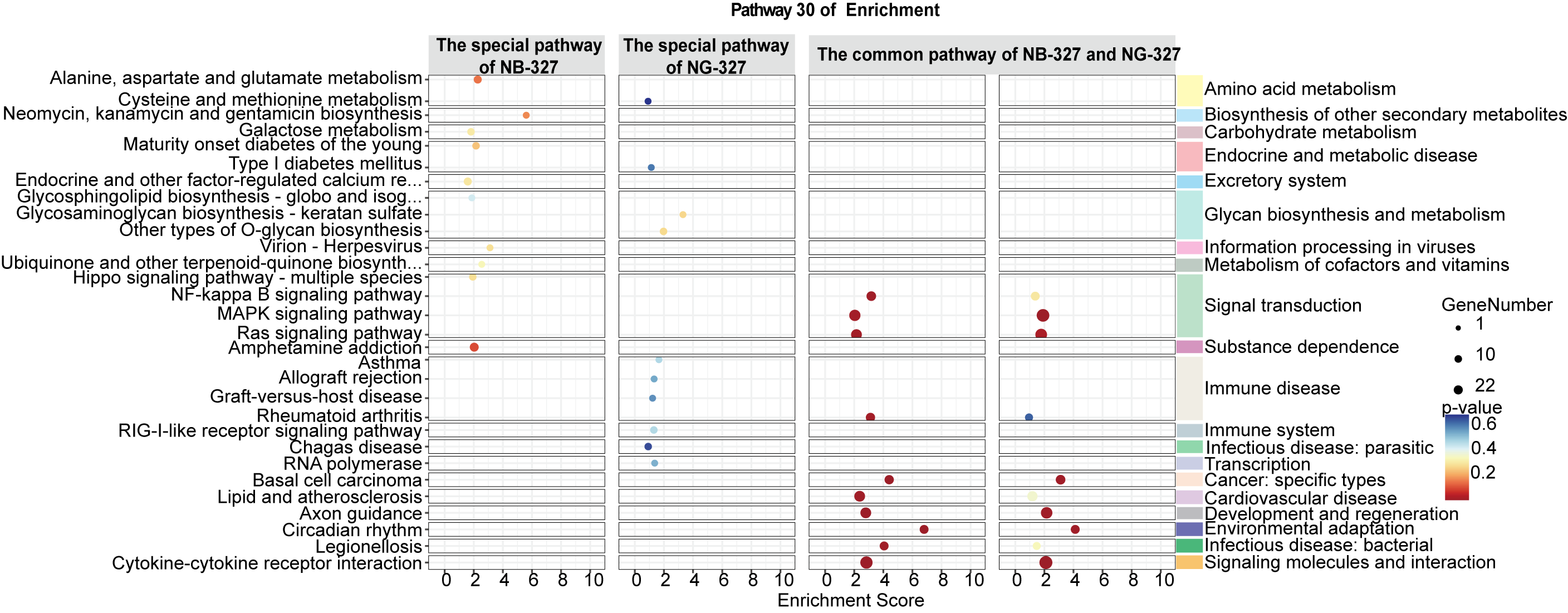

Supplement: Supplemental Information 5 — KEGG pathway enrichment analysis the overlapping and unique top 10 significantly enriched pathways in the NB-327 and NG-327, each compared to the NC group. [file peerj-14-20899-s005.png]

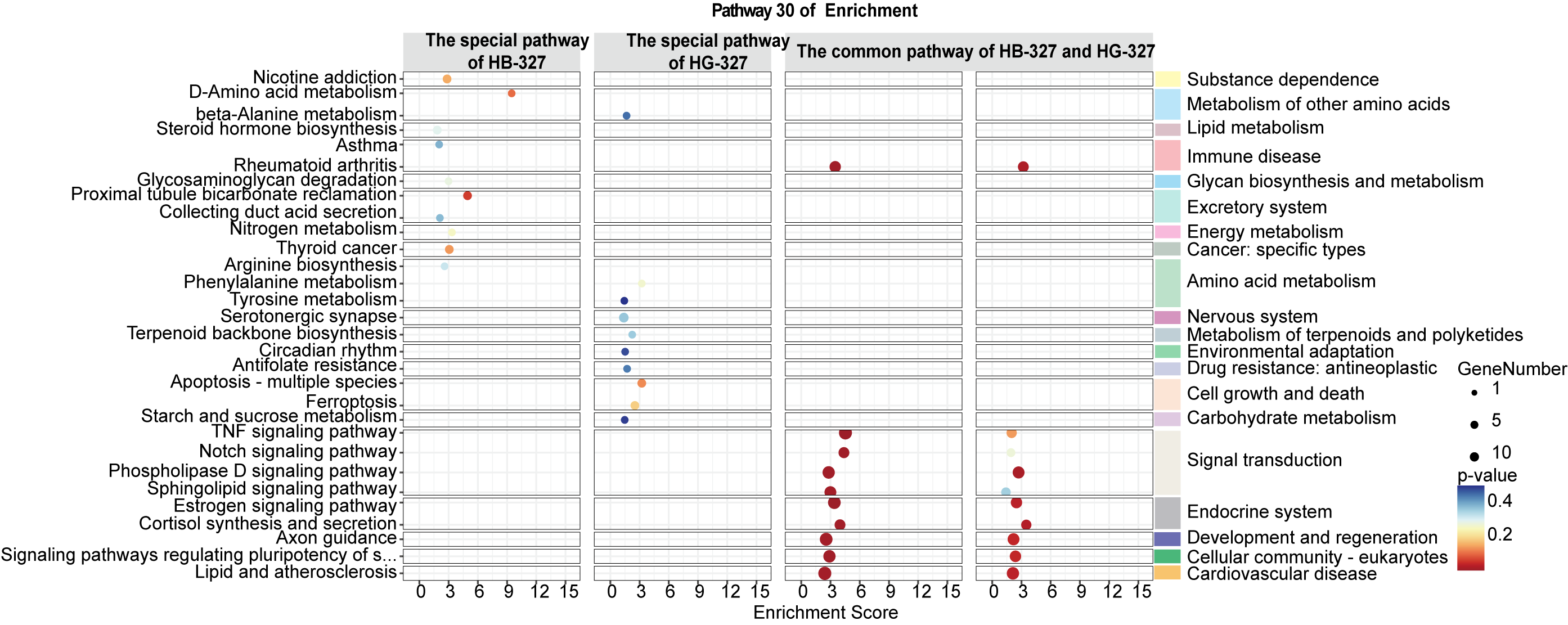

Supplement: Supplemental Information 6 — KEGG pathway enrichment analysis the overlapping and unique top 10 significantly enriched pathways in the HB-327 and HG-327, each compared to the NC group. [file peerj-14-20899-s006.png]

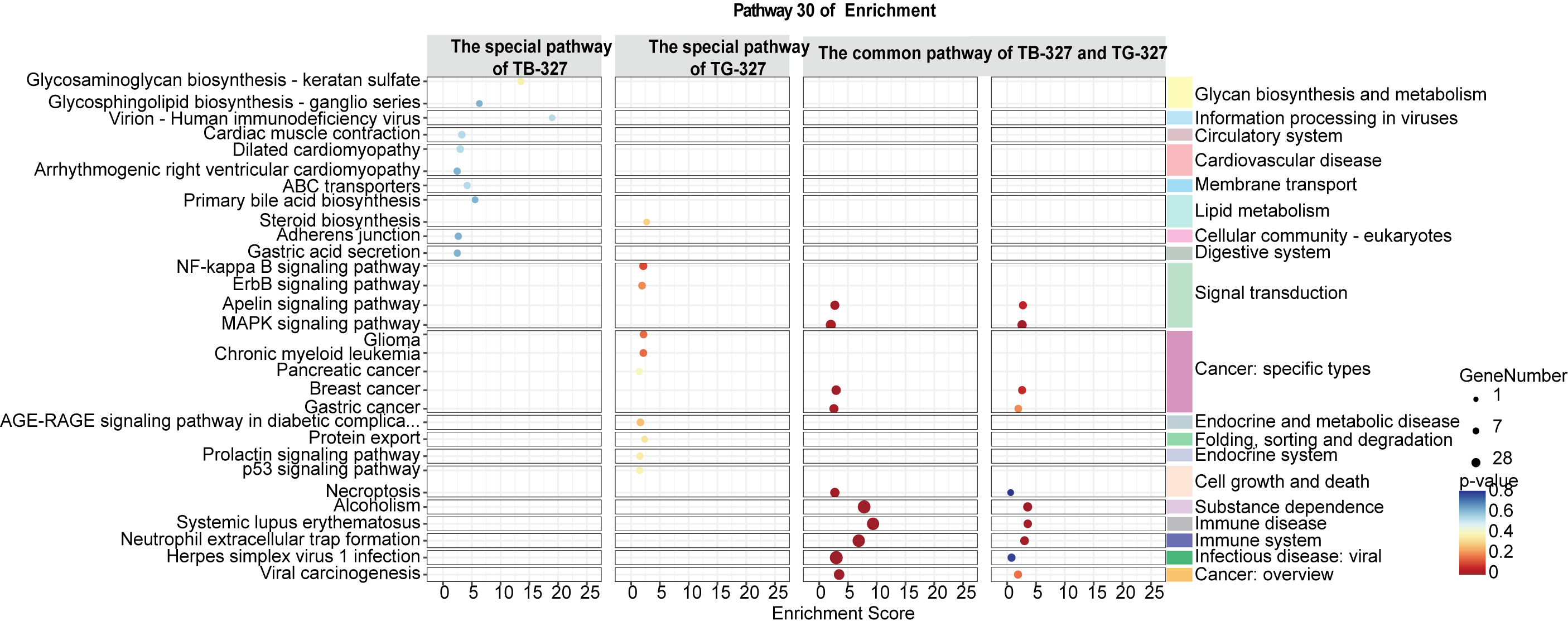

Supplement: Supplemental Information 7 — KEGG pathway enrichment analysis the overlapping and unique top 10 significantly enriched pathways in the TB-327 and TG-327, each compared to the NC group. [file peerj-14-20899-s007.png]

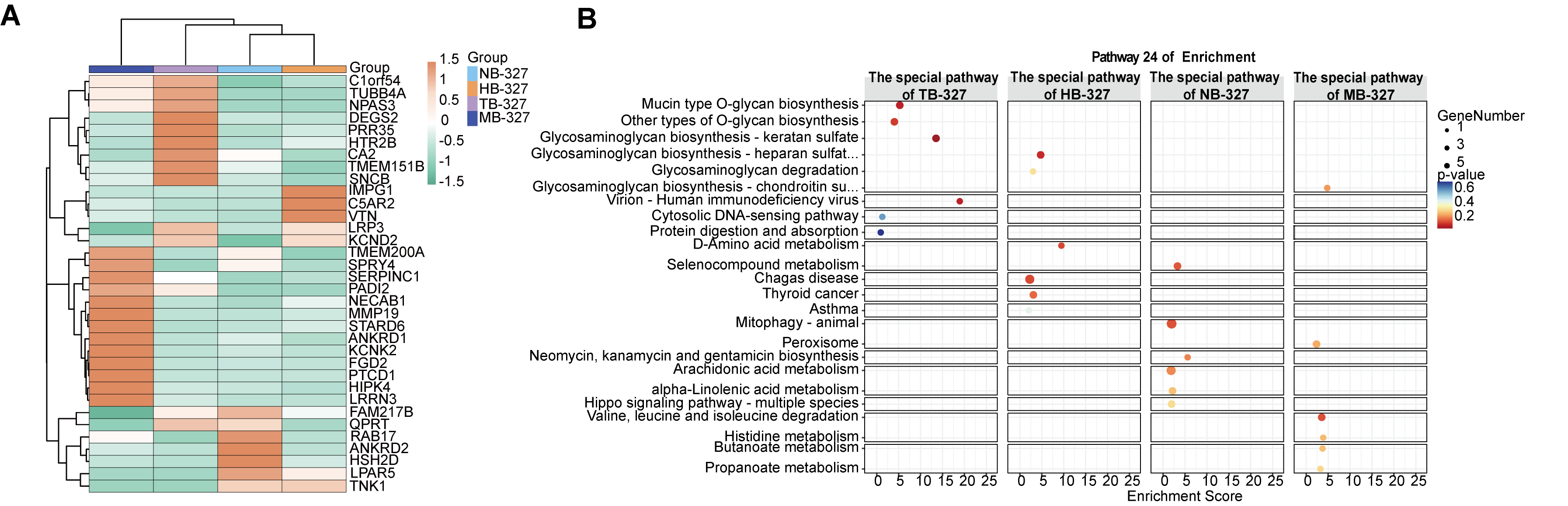

Supplement: Supplemental Information 8 — (A) Heatmap showing the expression of significantly differentially expressed genes in MB-327, NB-327, HB-327, and TB-327, with red indicating upregulated genes and blue indicating downregulated genes. (B) KEGG pathway enrichment analysis the unique top 6 significantly enriched pathways in the MB-327, NB-327, HB-327 and TB-327, each compared to the NC group. [file peerj-14-20899-s008.png]

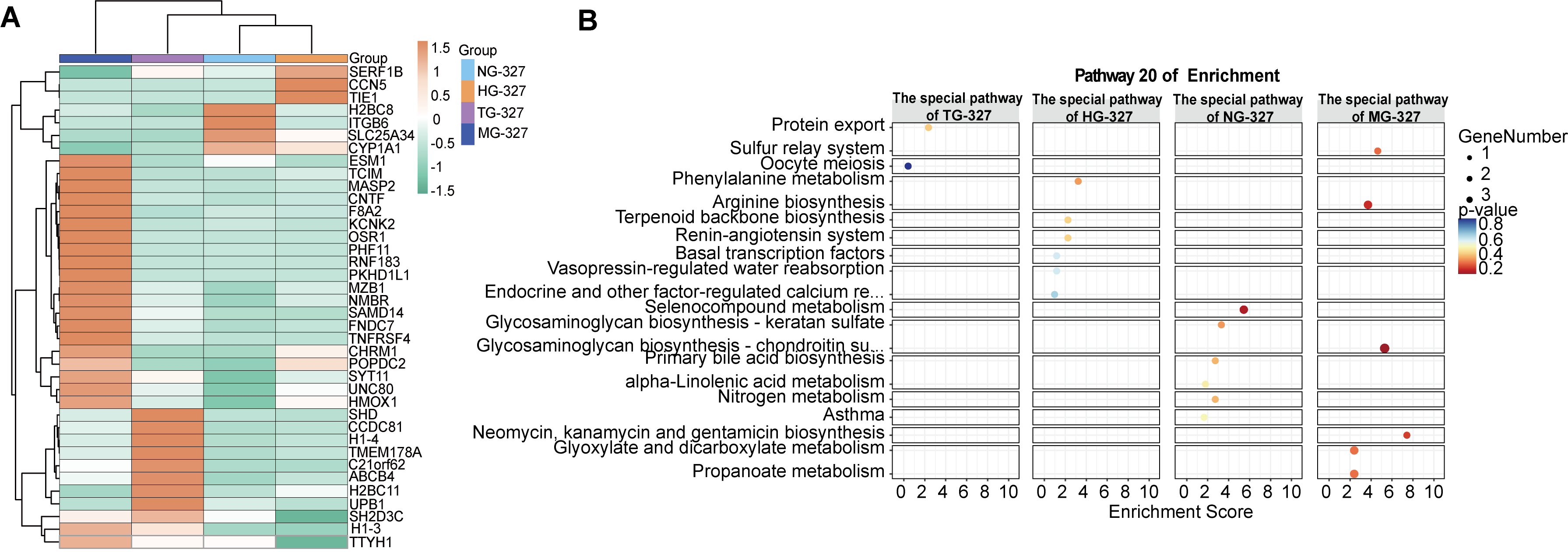

Supplement: Supplemental Information 9 — (A) Heatmap showing the expression of significantly differentially expressed genes in MG-327, NG-327, HG-327 and TG-327, with red indicating upregulated genes and blue indicating downregulated genes. (B) KEGG pathway enrichment analysis the unique top 5 significantly enriched pathways in the MG-327, NG-327, HG-327 and TG-327 groups, each compared to the NC group. [file peerj-14-20899-s009.png]
